# Simulation-Based Evaluation of Methods, Data Types, and Temporal Sampling Schemes for Detecting Recent Population Declines

**DOI:** 10.1093/icb/icac144

**Published:** 2022-09-14

**Authors:** Brendan N Reid, Malin L Pinsky

**Affiliations:** Department of Ecology, Evolution, and Natural Resources, Rutgers University, New Brunswick, NJ 08901, USA; Department of Ecology, Evolution, and Natural Resources, Rutgers University, New Brunswick, NJ 08901, USA

## Abstract

Understanding recent population trends is critical to quantifying species vulnerability and implementing effective management strategies. To evaluate the accuracy of genomic methods for quantifying recent declines (beginning <120 generations ago), we simulated genomic data using forward-time methods (SLiM) coupled with coalescent simulations (msprime) under a number of demographic scenarios. We evaluated both site frequency spectrum (SFS)-based methods (momi2, Stairway Plot) and methods that employ linkage disequilibrium information (NeEstimator, GONE) with a range of sampling schemes (contemporary-only samples, sampling two time points, and serial sampling) and data types (RAD-like data and whole-genome sequencing). GONE and momi2 performed best overall, with >80% power to detect severe declines with large sample sizes. Two-sample and serial sampling schemes could accurately reconstruct changes in population size, and serial sampling was particularly valuable for making accurate inferences when genotyping errors or minor allele frequency cutoffs distort the SFS or under model mis-specification. However, sampling only contemporary individuals provided reliable inferences about contemporary size and size change using either site frequency or linkage-based methods, especially when large sample sizes or whole genomes from contemporary populations were available. These findings provide a guide for researchers designing genomics studies to evaluate recent demographic declines.

## Introduction

Human impacts on wild populations have steadily intensified over the course of the Holocene, and multiple lines of evidence suggest the current era is heading towards a mass extinction event (Ceballos et al. 2015). For species of conservation concern, understanding recent population trends (on the scale of the past several decades or centuries) is critical to quantifying species vulnerability and implementing effective management strategies. Rapidly decreasing population sizes increase the risk of local extirpation or complete extinction in the near future (Caughley 1994). Populations with small population sizes are also at risk of losing genetic diversity and adaptive potential due to genetic drift (Franklin 1980, Lande and Barrowclough 1987) and can experience declining average fitness due to inbreeding depression (Hedrick and Kalinowski 2000, Keller and Waller 2002). Accurately estimating current and past population size is therefore a priority for conservation biologists and managers. Unfortunately, baseline data on census population sizes (*N*_c_) over the past few centuries are often missing or unreliable for species of conservation concern (Alagona et al. 2012, McClenachan et al. 2012).

Genomic methods offer a promising alternative to direct census data for inferring effective population size (*N*_e_) over time. *N*_e_ determines the rate of inbreeding and genetic drift over time, and although it is not directly substitutable for *N*_c_ (usually, *N*_e_ is <*N*_c_, sometimes by several orders of magnitude), it is an important determinant of the rate of evolutionary change (Waples 2022). Changes in *N*_e_ leave an imprint on genomic diversity within a population in two important ways. First, the demographic history of a population influences the distribution of allele frequencies in that population, also known as the site frequency spectrum (SFS). A population undergoing a demographic expansion, for example, will contain more rare alleles relative to a population with a constant size, while a population undergoing a demographic decline will contain fewer rare alleles (Griffiths and Tavaré 1998, Beichman et al. 2018). Because of this connection between the distribution of allele frequencies and demographic history, the expected SFS can be constructed for a given population history using coalescent simulations in which the probability of sampled individuals sharing a common ancestor is determined by the population size over time (Excoffier et al. 2013). Computationally efficient approximations of coalescent expectations can be obtained using diffusion models (Gutenkunst et al. 2009) or stochastic models (Kamm et al. 2020) which use continuous time rather than discrete generations, and population parameters can be estimated based on the observed SFS using likelihood methods. The effect of demographic change on the SFS accumulates over time and is dependent on both the mutation rate and the coalescence rate. This means that the signal of ancient demographic processes is easier to detect than the signal of recent change, especially in large populations where coalescence is less frequent, and larger sample sizes will be necessary to detect recent changes (Beichman et al. 2018).

Importantly, SFS methods assume that the loci used to construct the SFS are independent and unlinked. Nonrandom associations between loci can occur for multiple reasons, including physical linkage among loci on the same chromosome and genetic drift in finite populations (Hill 1981). The latter means that patterns of linkage disequilibrium (LD) across loci will also be shaped by demographic history. Multiple methods have been developed to infer population size from patterns of LD. For a sample of physically unlinked loci, LD should be close to zero in an infinite population, and the amount of “excess” LD can be used to estimate *N*_e_ at a particular time point. This method is most accurate when the population size is small and the sample size is large (close to the true *N*_e_; Waples 2006). For loci inherited on the same chromosome, the frequency of recombination and thus the amount of LD depends both on cumulative genetic drift and the frequency of recombination between the two loci, meaning that LD for loci with different linkage distances will reflect population size at different points in that population's history. Recombination frequency will usually increase with increasing physical distance on the chromosome, although recombination rate can vary throughout the genome (Peñalba and Wolf 2020). Thus, the pattern of LD across the genome, combined with a linkage map of known recombination rates, can be used to infer changes in *N*_e_ over time (Hayes et al. 2003). Recombination events are more frequent for loci with weaker physical linkage, and since recombination rates for weakly linked loci can be much higher than mutation or coalescence rates, LD data potentially contain more information for inferring recent changes in population size than SFS data alone (Hayes et al. 2003, Santiago et al. 2020). Critically, obtaining detailed linkage information requires the existence of an accurate reference genome for the organism of interest, which may not be available in many cases.

One promising avenue for inferring recent changes in *N*_e_ is by comparing genetic patterns in historical and modern samples. Advances in obtaining genetic material from museum specimens or other historical samples have made the acquisition of both baseline and contemporary genetic data (henceforth “temporal data”) a possibility (Nielsen and Hansen 2008, Bi et al. 2013, Habel et al. 2014, Diez-del-Molino et al. 2018, Oosting et al. 2019). For example, temporal RADseq data from salamanders has been used to accurately reconstruct known recent declines and expansions (Nunziata et al. 2017). In widespread species, such as Atlantic salmon, genomic signatures of population decline from targeted sequencing of historical and contemporary samples have also been used to identify which populations have recently declined and to infer the drivers of these declines (Lehnert et al. 2019).

In addition to temporal sampling, the type of genomic data and the availability of reference genomes can also influence the quality of inference for *N*_e_. Conservation genomics practitioners often use techniques that sample a moderate number of loci across the genome, such as RADseq (Andrews et al. 2016) or targeted sequencing (Meek and Larson 2019). Without a reference genome, these data can provide information on the SFS but are anonymous with regard to linkage information. Whole-genome sequencing is now within reach as well and can greatly increase the scope and precision of inference possible from conservation genomic studies (Brandies et al. 2019). Chromosome-level assemblies are the gold standard for providing linkage information; however, draft genomes with incomplete linkage information may be sufficient for making demographic inferences with some methods (Patton et al. 2019).

While several recent studies have used simulations to compare performance of methods for inferring recent population history under a given sampling scheme for either RADseq (Nunziata and Weisrock 2018) or whole-genome data (Patton et al. 2019), the amount of precision gained by adding historical genomic data compared to using only contemporary data remains unclear. The existing simulation studies have also examined performance under somewhat limited ranges of past and present population sizes, timings of population decline, and generation times. Failure to account for ancient population events, such as Pleistocene expansion or contraction, may also affect inferences made under SFS methods (Momigliano et al. 2021, Hoey et al. 2022). The lingering uncertainty from all of these potential sources can make it difficult for researchers to make objective decisions regarding how to best spend limited research funds to generate data that will yield the highest-quality inferences regarding recent demographic history.

To help guide study design for researchers interested in recent demographic inference using genomic data, we compare here the performance of four inference methods and three temporal sampling schemes across simulated reduced-representation and whole-genome datasets representing scenarios of recent population stability or decline. We aim to answer the following primary questions: (1) Which methods provide the most accuracy and precision for identifying population declines using contemporary data? (2) How do historical genomic data alter the accuracy of demographic inference? By evaluating the accuracy of different study designs and inference methods, we provide concrete recommendations for conservation biologists interested in reconstructing the recent demographic history of a diverse array of potential study organisms.

## Methods

### Study outline

To evaluate different methods for estimating contemporary changes in population size, we simulated whole genomes from populations with known histories representing either stability or decline over the past 200 years. We then subsampled these genomes to generate a number of reduced datasets incorporating a smaller number of individuals and/or a random subset of loci distributed throughout the genome. Finally, we applied four estimation methods that have been developed to infer recent declines. Each estimation method can accommodate different data types and temporal schemes and takes different inputs (Table 1). We compared power to detect declines as well as the accuracy and precision for each method.

**Table 1:** Characteristics of software used to infer recent declines in this study. SFS = site-frequency spectrum, LD = linkage disequilibrium, WGS = whole-genome sequencing, RAD = restriction-associated DNA or similar reduced-representation data, VCF = variant call format file.

Software	Inference method	Data type	Temporal scheme	Input format	Additional inputs
momi2	SFS	WGS or RAD	Contemporary or temporal	SFS/VCF*	Mutation rate, generation time
Stairway Plot	SFS	WGS	Contemporary only	SFS/VCF*	Mutation rate, generation time
NeEstimator	LD	RAD	Temporal	Genepop	Chromosome locations (optional)
GONE	LD	WGS	Contemporary only	PLINK	Recombination map (optional)

* Note that the SFS-based methods accept an SFS for their input but that this SFS can be calculated from a standard VCF file.

### Simulation scope and approach

We consider a single panmictic population of diploid, dioecious organisms sampled at a contemporary time point—or zero years before present (ybp)—and at several time points in the recent past (t = 120, 90, 60, and 30 ybp) corresponding to samples that could be represented in natural history collections or genetic monitoring programs. We express time in years rather than generations so that we can examine more complex demographic scenarios, such as overlapping generations. We do not consider “paleogenomes” from the more distant past in this paper.

**Fig. 1.**
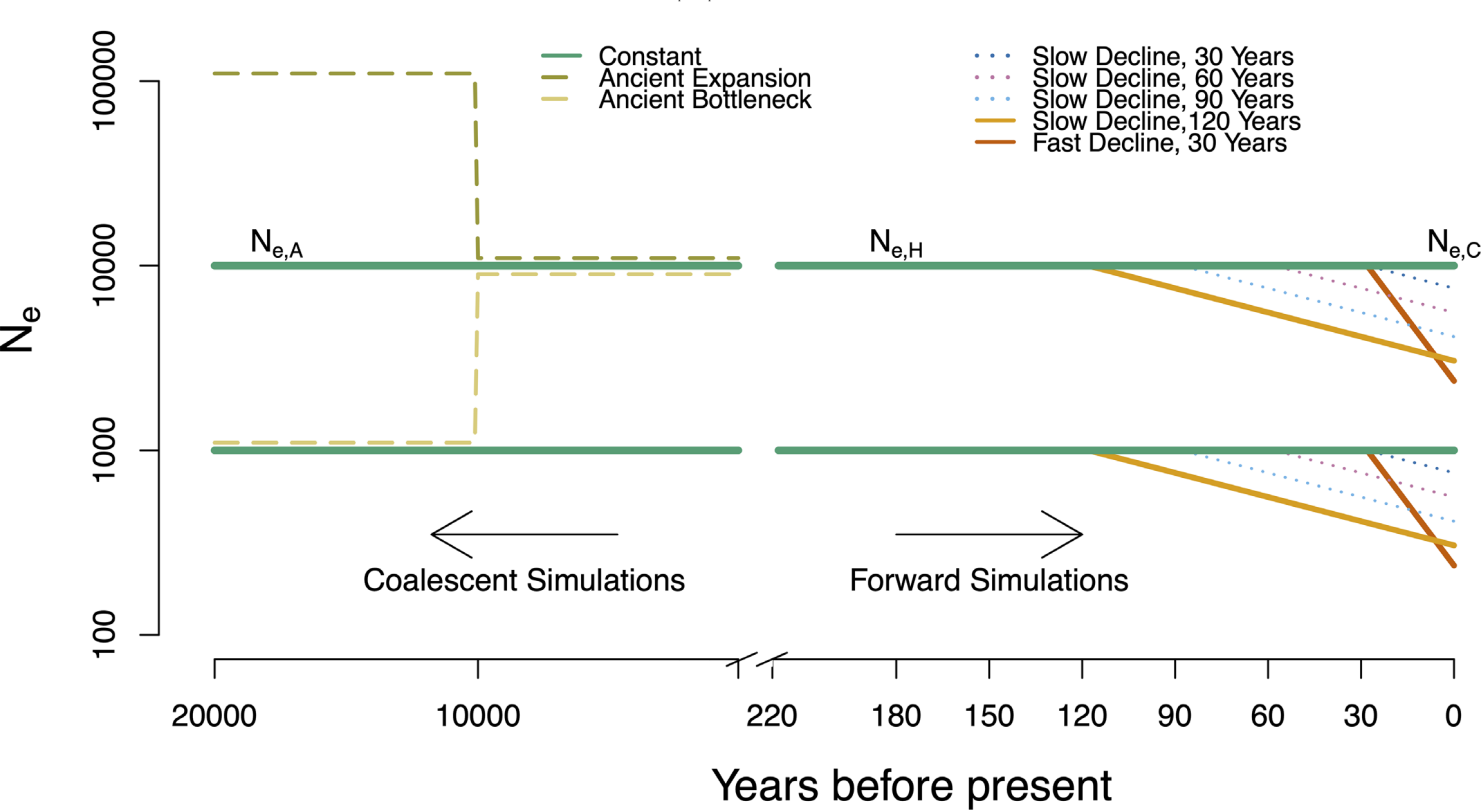
Simulation schemes. Parameters of interest are effective population sizes at three time points: contemporary effective population size (N_e, C_), historic effective population size (N_e, H_), and ancient effective population size (N_e, A_). Solid lines represent simulation scenarios used for mean error calculations (constant size over recent time, a fast decline beginning 30 ybp, and a slow decline beginning 120 ybp). Accuracy and bias were also assessed for three other recent decline scenarios (dotted lines) and two ancestral population size change scenarios (dashed lines).

We simulate data from this population in two distinct stages (Fig. 1). The first stage uses demographically realistic forward-time simulations to generate a set of recent genealogies. The sex of each individual in the population was randomly determined (50/50 chance of being male or female, expected sex ratio = 0.5). Population size in forward simulations was regulated by controlling the number of offspring (*N*_O_) generated in each time step. At each time step, *N*_O_ offspring were generated by randomly selecting one male and one female parent with replacement from all breeding-age individuals for each offspring.

We considered two different life history patterns in these simulations. The first pattern (G1) represented an annual organism with a generation time of one year and no overlapping generations (all individuals in the population can breed and all die upon reaching an age of one year). The second (G2) pattern represented an organism with overlapping generations, with age at first breeding equal to one year, an age-specific mortality probability, and a maximum age equal to eight years. The mortality probabilities and age at first breeding were set such that the mean age of a breeding individual (and thus the mean generation time) was ∼3 years and the expected number of breeding-age individuals (*N*_B_) was equal to *N*_O_ in a stable population. Since for each simulated situation the number of offspring in a given time step equaled the number of breeding age individuals, regardless of population size, the distribution of reproductive success approximately followed a Poisson distribution as expected in an ideal population with random mating, with each parent contributing genes to a mean of two offspring and the expected variance in offspring *N*_O_ being approximately two. *N*_O_ in each generation should therefore be roughly equivalent to *N*_e_ for both life history patterns (Hill 1972).

The forward simulations began 100 years before the first historical sampling time point (i.e., 220 ybp). For the baseline simulations, we set the initial population size (*N*_e, H_) to either 1,000 or 10,000. *N*_O_ in each subsequent generation either remained stable or began an exponential decline (with λ = *N*_O_ in the current year / *N*_O_ in the following year) at a time point directly after one of the historical sampling points (*T*_dec_ = 120, 90, 60, or 30 ybp), eventually reaching a contemporary effective population size (*N*_e, C_) at the final time point (zero ybp). For all declining populations we conducted a set of simulations with λ = 0.99, resulting in *N*_e, C_/*N*_e, H_ of 0.74, 0.55, 0.40, and 0.30, respectively for each decline scenario. We also conducted one simulation with a recent decline (*T*_dec_ = 30) with λ = 0.95 and *N*_e, C_/*N*_e, H_ = 0.21. This higher λ value was only paired with a recent decline because rapid declines beginning earlier resulted in extremely small population sizes.

We conducted five iterations of each forward demographic simulation using SLiM v.3.3.2 (Haller and Messer 2019) and recorded the full pedigree as well as the number of breeders of each sex for each iteration. We then simulated 25 “chromosomes” for each demographic iteration by conducting an independent simulation (with the pedigree fixed to the recorded pedigree) of a single sequence of length 30 Mb and a per-generation recombination rate of 10^−8^ per base per generation (or 1 centiMorgan (cM)/Mb, which is within the range of recombination rates observed for plants and animals; Stapley et al. 2017). To decrease the computational intensity of chromosome simulations, we did not simulate mutations in SLiM, instead using tree sequence recording and coalescent simulations (see below) for generating polymorphisms (Haller et al. 2019). For tree sequence recording, we recorded 200 individuals at each potential *T*_dec_ as well as all individuals at 0 ybp.

The second stage of simulations involved simulating genetic data for these genealogies using reverse-time coalescent methods. Simulations were performed using msprime v.0.7.4 (Kelleher and Lohse 2020) in the python package pyslim v.0.501, and populations were projected backward for a number of generations sufficient for all sampled individuals to reach a common ancestor using a coalescent process (i.e. without incorporating the complex life history used in forward-time simulations for G3). The effective population size at the initiation of the simulation was set to the number of breeding-age individuals in the first generation of the forward-time simulation (*N*_e, H_). Populations in the coalescent simulations either remained stable over time or experienced a 10-fold size change (representing either an ancestral expansion or an ancestral bottleneck) at 10,000 generations before present, and they remained at this ancestral population size (*N*_e, A_) for the remainder of the simulation (until all loci reached coalescence). Ancestral bottlenecks and expansions were only simulated for populations with a larger historic population size (*N*_e, H_ = 10,000), for the G1 life history pattern, and for a restricted set of recent demographic scenarios (constant population size, a rapid decline starting 30 ybp, and a slow decline starting 120 ybp). Eighteen demographic scenarios in total were simulated. We simulated data with a recombination rate of 10^−8^ as used in the forward simulations, and we added mutations to simulated chromosomes using a per-generation, per-base mutation rate of 10^−8^ (within the range of mutation rates observed for plants and animals; Lynch 2007). From these two simulation stages, we generated VCF files containing all variable sites for each simulated chromosome from 200 randomly selected individuals at each time point.

### Sampling designs

We subsampled from these full datasets to represent realistic constraints of study design choices. We generated datasets with total sample sizes *n* of 20, 50, 100, and 200 individuals. Temporal sampling schemes can range from a single comparison between a historic baseline and a contemporary sample to a number of samples collected over several time points, as in fisheries monitoring (Hutchinson et al. 2003) and repeated museum collections (Gauthier et al. 2020). As such, for each dataset samples were either all collected from the contemporary timepoint (*n* samples at 0 ybp; contemporary-only dataset), split evenly between a contemporary and a baseline timepoint (*n*/2 samples at 0 ybp and *n*/2 samples at 120 ybp; two-sample dataset), or split between five serial timepoints (*n*/5 samples at 0, 30, 60, 90, and 120 ybp, respectively; serial dataset). For whole-genome datasets we used the three smaller sample sizes (total *n* = 20, 50, or 100). To create RADseq-like datasets, we used the three larger sample sizes (total *n* = 50, 100, or 200) and applied an additional filter to keep only SNPs found within a set of randomly placed 150bp loci on each of the 25 chromosomes. For RADseq-like datasets, we used either 400 RADseq loci per chromosome (10,000 total loci) or 2000 loci per chromosome (50,000 total loci). We generated and conducted inference on 2430 simulated datasets in total.

### Inference on simulated datasets

We applied four different inference methods to the simulated datasets. The methods chosen represent commonly used software packages that use either the SFS or LD to infer current and past population sizes and can incorporate either temporal data, whole-genome data, or both. For conducting inference with temporal data using the SFS, we used the program momi2 (Kamm et al. 2020), a model-based method for demographic inference that can incorporate whole-genome or RAD data. We used pyslim to compute allele counts for each chromosome at each time point, and we combined these counts into aggregate folded SFS for each time point. momi2 assumes a branching tree-like population structure, and to accommodate sampling multiple time points from a single continuous population in momi2, we specified that each SFS was sampled as a “leaf” from a branch at its corresponding sampling time, after which all lineages from that population were shifted to a new branch from which the next sample was taken. For whole genome data, the total number of sites was set to the genome size (750Mb), while for RADlike data the total number of sites was set to 150 times the number of loci (1.5 Mb or 6 Mb) to represent 150 bp RAD loci. For each simulated dataset we fit four different demographic models: (1) a model with a single constant population size parameter, *N_constant_*; (2) a model with two population size parameters (size at 0 ybp, *N_contemp_*, and at 120 ybp, *N_historic_*) and a time parameter specifying the time at which the population began an exponential size change (*T_rc_*); (3) a model with an instantaneous ancient size change $\hat N$_ancient_ occurring at *T_ac_*; and (4) a model including both the ancestral and recent size changes. Potential ranges for *N_constant_, N_contemp_, N_historic_*, and *N_ancient_* were set to 10 – 500,000 individuals (the size change was not assumed to be a decline). The range for *T_rc_* was set to 10–120 ybp and the range for *T_ac_*was set to 1,000–100,000 ybp. The rate of exponential size change was fully determined by the time of decline and the two population size parameters. We did not constrain recent size changes to be declined, and as such we evaluated whether momi2 inferred the population to be either stable, declining, or expanding. We fit all models to each simulated dataset using the Truncated Newton (TNC) optimizer, and we recorded all parameter estimates as well as the likelihood and AIC for each model. We retained parameter estimates for population size at 0 ybp $\hat N$_e, H_ and 120 ybp $\hat N$_e__, C_ for the model with the lowest AIC for each dataset.

For conducting inference using the SFS with whole-genome data, we used Stairway Plot 2 (Liu and Fu 2020). We used the vcf2sfs script (https://github.com/shenglin-liu/vcf2sfs) to compute the folded SFS input. We set the total number of sites (including monomorphic sites) to 750Mb, the number of random breakpoints for each iteration to 7, 15, 22 , and 28, the mutation rate per generation to 1 × 10^−8^, and the generation time to 1 year, and we used 67% of the sites for training. We retained the most recent median estimate of population size as $\hat N$_e, C_. As the time bins for Stairway Plot estimates can be somewhat irregularly spaced, we used the median population size estimate for the time bin closest to 120 ybp that was closest to this time point a $\hat N$_e, H_, and we used the 2.5% and 97.5% estimates as confidence intervals (CIs).

For conducting inference based on excess LD using RAD-like data, we used NeEstimator2 (Do et al. 2014). The LD method outperformed two other methods implemented in NeEstimator in a previous study (Gilbert and Whitlock 2015). We only used the two-sample scheme for assessing performance of NeEstimator since contemporary-only sampling does not allow for inference of historic size with this method. Before running, we converted vcf files to genepop files using the vcf2genepop.pl script (https://github.com/z0on/2bRAD_denovo/blob/master/vcf2genepop.pl). We used an allele frequency cutoff of 0.05 and assumed random mating. We used the point estimates and the jackknife 95% CIs for population size at 0 ybp and 120 ybp as $\hat N$_e, C_ and $\hat N$_e, H_, respectively.

For conducting inference using LD with whole-genome data, we used GONE (Santiago et al. 2020). We used plink (Purcell et al. 2007) to convert the vcf file to ped/map format. We ran GONE using the default parameters (unknown phase, 1 cM/Mb, Haldane correction, 2000 generations, 400 bins, MAF = 0, allowing SNPs with zeroes, using all chromosomes, 50,000 SNPs/ chromosome, hc = 0.05, 40 reps, and 20 threads). We used the estimates for population size at 0 ybp and 120 ybp as $\hat N$_e, C_ and $\hat N$_e, H_, respectively. After Santiago et al. (2020), we performed resampling to estimate a CI for $\hat N$_e, C_ and $\hat N$_e, H_. Since some datasets contained a relatively small number of SNPs) <300,000 SNPs compared to the datasets in Santiago et al. (2020), we took a random sample of 50,000 SNPs 40 times and re-ran the program to generate 95% CIs.

### Effects of genotyping errors and allele frequency filters

Some demographic inference methods (including momi2) assume no errors in genotyping and no filtering of genotypes based on allele frequency. Since these conditions are rarely met in practice for non-model species, we performed additional inferences using modified datasets to explore the effects of some potential violations of these assumptions on inference accuracy.

Genotyping errors are more likely to occur for historical samples due to lower coverage and postmortem DNA damage in older samples. For example, Bi et al. (2013) found error rates that were almost five-fold higher (0.19%) in historical samples compared to contemporary error rates (0.04%). Genotyping errors are most likely to create singleton SNP genotypes that would potentially impact SFS-based analyses. Minor allele frequency filters are commonly applied to SNP datasets; while this may improve inference for some applications, such as assessing population structure using STRUCTURE-like methods (Linck and Battey 2019), distorting the SFS by removing low-frequency alleles could also negatively impact other analyses (Lou et al. 2021). To assess the potential effect of errors in historical genotypes, we added singletons to the SFS in momi2 at three different rates (1e^−3^, 1e^−4^, or 1e^−5^ singletons per site) for RAD-like datasets and re-ran the momi2 inferences. We also applied a minor allele frequency filter of 0.01 to the data and re-ran momi2 inferences as well.

### Evaluating methods

For datasets simulated with a recent decline, we considered the decline to be correctly inferred when a model including a recent decline had the lowest AIC and $\hat N$_e, C_ < $\hat N$_e, H_ (for momi2) or when the upper 95% CI for $\hat N$_e, C_ was lower than the lower 95% CI for $\hat N$_e, H_ (for the other methods). For datasets simulated with constant population size, we considered the demographic history to be correctly inferred when a model including a constant recent population size had the lowest AIC and $\hat N$_e, C_ < $\hat N$_e, H_ (for momi2), or when the 95% CIs for $\hat N$_e,C_ and $\hat N$_e, H_ overlapped. Because StairwayPlot and NeEstimator provide 95% CIs for the contemporary and historic population sizes themselves, rather than the difference in population sizes, this test for a decline is somewhat overly conservative (Cumming and Finch 2005).

We evaluated and visualized error using two different approaches. For each method, we calculated mean absolute percentage error for $\hat N$_e, C_ and $\hat N$_e, H_ for each simulated dataset *i* over *n* total datasets using the following equation:

\begin{eqnarray*} \frac{{100}}{n}\mathop \sum \limits_{i{\rm{\,\,}} = {\rm{\,\,}}1}^n \frac{{\left| {inferre{d_i} - simulate{d_i}} \right|}}{{simulate{d_i}}}, \end{eqnarray*}

We calculated mean error on an aggregated subset of scenarios, including three demographic scenarios (constant population size, a rapid decline starting 30 ybp, and a slow decline starting 120 ybp), a generation time of 1, and both initial population sizes. We calculated mean error separately for the WGS and 50k RAD loci datasets.

Since this metric uses absolute value and does not convey potential directional biases, we also visualized concordance between true and simulated values by plotting the log_10_ ratio of the inferred to the simulated value across all simulated demographic scenarios.

For two methods, we assessed alternate metrics of accuracy. Since we fit multiple alternate models in momi2, we evaluated how often the correct model (constant population size for data generated under the constant demographic model, or size change for data generated under the size change model) had the best support (defined as the model with the lowest AICc). Since NeEstimator can return estimates of “infinity” in some situations, we also identified the proportion of simulations for which this occurred for $\hat N$_e, H_ and $\hat N$_e, C_. Due to a large number of “infinite” estimates from NeEstimator at *n* = 50 (Supplementary Fig. 1), we excluded this sample size from mean error calculations.

## Results

### Power to detect declines

**Fig. 2.**
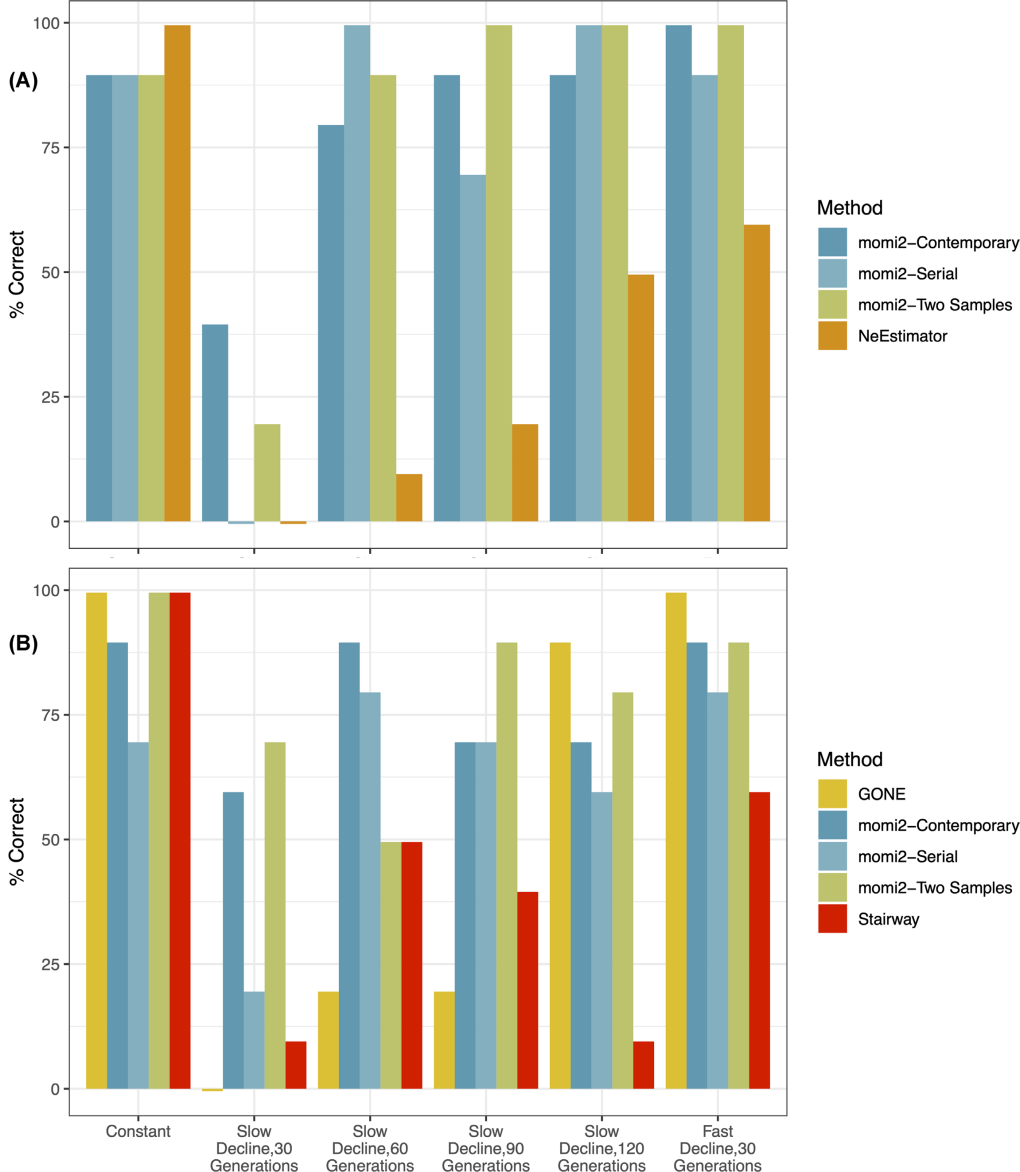
Power for detecting the correct demographic scenario for (A) RAD data and (B) WGS data. Results are shown for the largest sample size for each data type (RAD *n* = 200, WGS *n* = 100).

Overall, momi2 and GONE exhibited the highest power to correctly detect or reject recent declines. With large sample sizes (*n* = 200) and RAD-like data, momi2 correctly identified declines for ≥80% of simulated datasets and only performed poorly when declines were recent (30 generation ago) and slow (λ = 0.99) (Fig. 2A). momi2 did not perform as well using WGS data, although it was still ≥80% accurate for detecting slow declines ≥90 generations (Fig. 2B). Decreasing samples sizes for momi2 generally decreased the power to detect declines (Fig. 2, Supplementary Table 1, Supplementary Table 2). GONE exhibited ≥90% accuracy for detecting more severe declines (λ = 0.99 for 120 generations or λ = 0.95 for 30 generations) with large samples sizes (*n* = 100). Power to detect declines with GONE decreased for lower sample sizes and for less severe declines (Fig. 2B). The other methods (Stairway Plot and NeEstimator) generally had lower power compared to momi2 and GONE for similar sample sizes (Fig. 2; Supplementary Table 1, Supplementary Table 2).

### Accuracy and bias for estimating *N*_e, H_ and *N*_e, C_

**Fig. 3.**
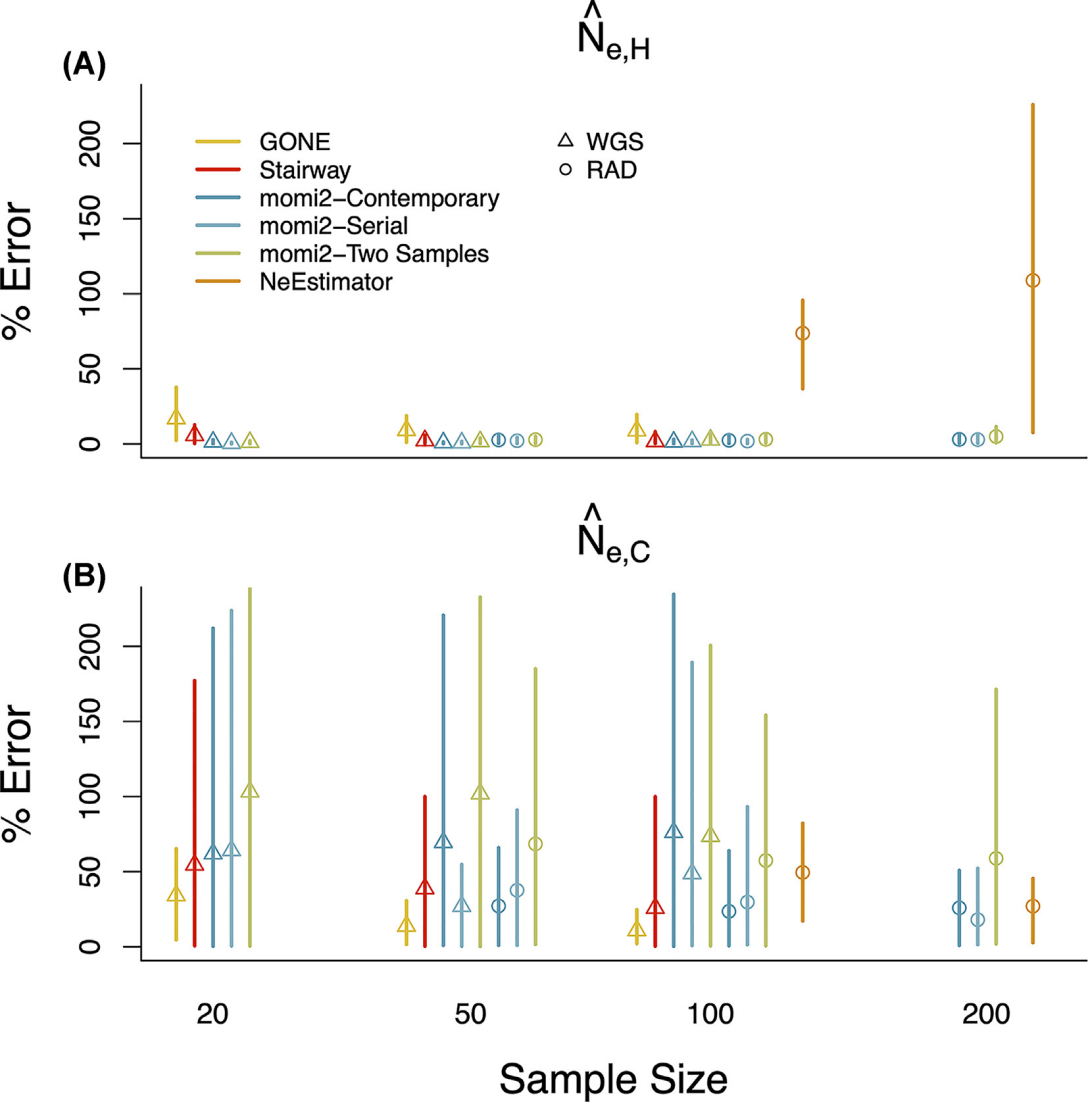
Mean absolute percentage error for estimating (A) historic effective population size (*N*_e, H_) and (B) contemporary effective population size (N_e, C_). Error bars show 10%–90% quantiles for each.

Mean absolute error for estimating *N*_e, H_ was the lowest for the SFS-based methods (momi2 and the Stairway Plot; Fig. 3A). Mean error for estimating *N*_e, H_ was somewhat higher for GONE, an LD-based method, than for the SFS-based methods, and the other LD-based method (NeEstimator) displayed substantially higher error for estimating *N*_e, H_ than any of the other methods (Fig. 3A). Mean absolute error tended to be substantially higher overall for estimating *N*_e, C_ (Fig. 3B) than for *N*_e, H_ across methods. GONE tended to have lower error for estimating *N*_e, C_ than SFS-based methods, while NeEstimator had comparable or higher error compared to momi2.

Mean absolute error for estimating *N*_e, H_ did not strongly depend on sample size for SFS-based methods (Fig. 3A). Mean error for estimating *N*_e, H_ decreased with sample size for GONE and actually increased for NeEstimator. For *N*_e, C_, on the other hand, mean error did tend to decrease with increasing sample sizes across methods (Fig. 3B).

Whole-genome and RAD data performed comparably for estimating *N*_e, H_ with SFS-based methods (Fig. 3A). There was no clear difference between the two data types for estimating *N*_e, C_ as well, and the most accurate estimates were produced by a WGS method (GONE with sample sizes 50–100) and an SFS method (momi2 with sample size of 200).

In general, there were no strong directional biases in estimating *N*_e, H_ except for NeEstimator with a sample size of 100, which produced downward-biased estimates (Supplementary Fig. 2a, Supplementary Fig. 3). Estimates of *N*_e, C_ tended to be biased upward when using a serial sampling scheme in momi2, for the rapid decline scenario in momi2, and for some scenarios for the Stairway Plot, but were otherwise fairly unbiased (Supplementary Fig. 2b, Supplementary Fig. 4).

### Generation time and accuracy

**Fig. 4.**
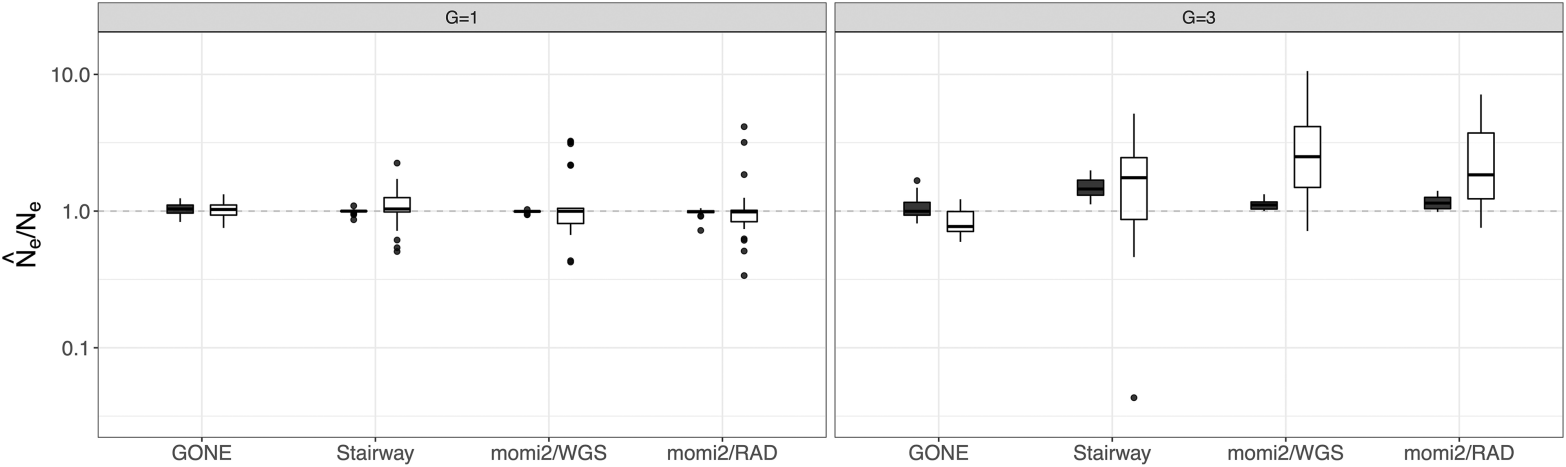
Relationship between generation time and accuracy and precision. Results are shown as box-and_whisker plots for the largest sample size for each data type and for inferences made using contemporary-only data. Results for *N*_e, H_ are shown in black and results for *N*_e, C_ are shown in white. Ratio of estimated to true *N*_e_ is plotted on a log_10_ scale. Perfect agreement between simulated and estimated values is shown as a 1:1 dotted line.

Accuracy of inferences for *N*_e, H_ based on simulations conducted using a generation time of three exhibited similar accuracy for momi2 and GONE overall compared to simulations conducted using a generation time of one (Fig. 4, Supplementary Fig. 6). For the Stairway Plot; however, *N*_e, H_ estimates for the longer generation time were less accurate and were biased upward. Estimates for *N*_e, C_ were biased slightly lower for a generation time of three but were otherwise fairly accurate for GONE between the two generation times. However, increasing generation time greatly reduced accuracy for the Stairway Plot and for momi2 when using contemporary-only data (Fig. 4; Supplementary Fig. 7).

### Effects of ancestral expansions and bottlenecks on accuracy and model selection

**Fig. 5.**
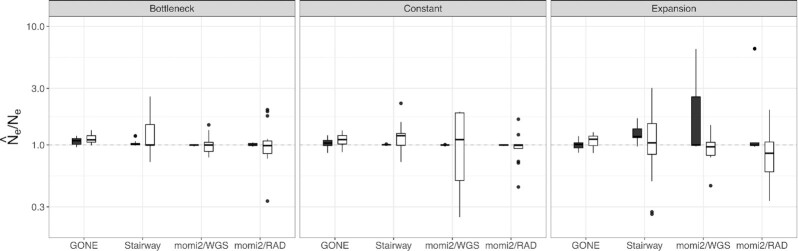
Effects of ancestral expansions and bottlenecks on accuracy and precision. Results are shown for the largest sample size for each data type and for inferences made using contemporary-only data. Results for *N*_e, H_ are shown in black and results for *N*_e, C_ are shown in white. Ratio of estimated to true *N*_e_ is plotted on a log_10_ scale. Perfect agreement between simulated and estimated values is shown as a 1:1 dotted line. Model selection results for these scenarios are shown in Supplementary Fig. 7.

Ancestral bottlenecks or expansions did not seem to strongly affect inferences of either *N*_e, H_ or *N*_e, C_ made with GONE (Fig. 5). For the SFS-based methods, ancestral bottlenecks did not affect estimates of *N*_e, H_, but ancestral expansions resulted in an upward bias for estimates of *N*_e, H_ for the Stairway Plot and in some iterations for momi2. Estimates of *N*_e, C_ made using whole-genome data with momi2 were somewhat more accurate when an ancestral bottleneck had occurred compared to the constant ancestral size scenario or ancestral expansion scenarios. For RAD data, accuracy for *N*_e, C_ was the highest for the constant size scenario and the lowest when an ancestral expansion had occurred. Model selection in momi2 using RAD data was most accurate for the ancestral bottleneck scenario and least accurate for the expansion scenario (Supplementary Fig. 8). For WGS data, momi2 again often selected the wrong model for the constant-size scenario with ancestral expansions or bottlenecks (Supplementary Fig. 8).

To explore the effects of model misspecification, we also used momi2 to fit models without ancestral size changes to data that did have these changes. In these cases, estimates of *N*_e, H_ were consistently biased either low (for the ancestral bottleneck scenario) or high (for the ancestral expansion scenario; Supplementary Fig. 9). For *N*_e, C_, accuracy was also reduced somewhat (particularly for the two-sample scenario) and resulted in a slight upward bias for the ancestral bottleneck scenario. In the case of the ancestral expansion scenario, model mis-specification did not affect inferences for serial sampling but did result in biases for the other scenarios, particularly the two-sample scenario (Supplementary Fig. 9).

### Effect of minor allele filtering and singleton errors on momi2

**Fig. 6.**
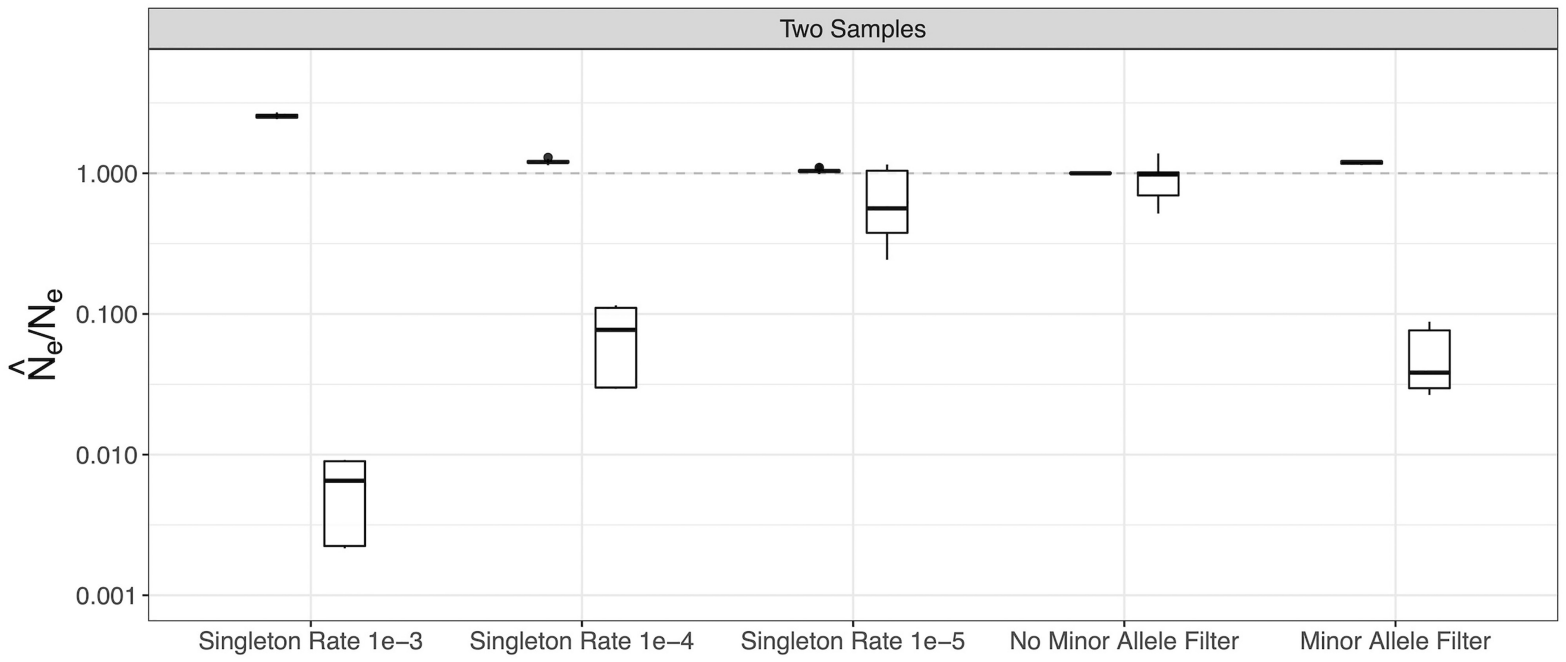
Effect of minor allele filtering and singleton errors on accuracy and precision for momi2 using two temporal samples. Results for *N*_e, H_ are shown in black and results for *N*_e, C_ are shown in white. Ratio of estimated to true *N*_e_ is plotted on a log_10_ scale. Perfect agreement between simulated and estimated values is shown as a 1:1 dotted line.

When using two-sample data in momi2, minor allele filtering introduced small upward biases in estimated *N*_e, H_, but a strong downward bias for *N*_e, C_ (Fig. 6). Adding singleton errors also introduced a small upward bias in *N*_e, H_ but had a much larger effect on *N*_e, C_, driving a strong downward bias at higher error rates (Fig. 6). Adding singleton errors to the contemporary-only dataset at the same rate as the two-sample dataset caused an extremely strong upward bias in estimates of both *N*_e, H_ and *N*_e, C_ (Supplementary Fig. 10).

### Performance of temporal sampling relative to contemporary-only sampling

Two-sample and serial sampling schemes did not show a consistent benefit over contemporary-only sampling in momi2, although these schemes did outperform contemporary-only sampling in certain cases. When using WGS data, serial and two-sample schemes performed better at identifying more recent slow declines than contemporary-only data (Fig. 2B). The two-sample scheme also performed better than the contemporary-only scheme when the generation time was 3 years (Supplementary Fig. 6). Finally, the bias produced by minor allele filtering was less pronounced for two-sample data and absent for serially sampled data (Supplementary Fig. 11).

## Discussion

Researchers interested in estimating population size change over recent time scales now have a larger selection of methodological tools and types of data available to them than ever before. Combined with limited resources, this can lead to difficult choices between generating more data from many contemporary individuals, obtaining historical and modern genomic data to provide a temporal comparison from fewer individuals, or generating whole-genome data and, potentially, a reference genome for their species of interest. While the suite of tools available is constantly expanding, the simulation-based analyses presented here provides guidance for such researchers regarding how certain types of data and analyses perform relative to others, and which analyses may be most robust to potential confounding factors.

### Inferring recent versus historical population sizes

Inferring historical changes in population size using modern methodologies has generally been considered an easier task than inferring recent changes, with the former possible based on a single whole genome (Li and Durbin 2011) or a reduced-representation data from a small number (>10) of individuals (Beichman et al. 2018). Our results supported this generalization for SFS-based methods. Inference using momi2 and the Stairway Plot both resulted in extremely low error rates regardless of sample size or data type (whole genome versus reduced representation), though with some caveats that incorrect model specification introduces biases with momi2. Among the linkage-based methods, GONE performed somewhat worse than either SFS-based method for inferring historic size, although performance improved at higher sample sizes and error was still fairly low (usually <25%).

In contrast to inference of historical population dynamics, Beichman et al. (2018) recommended avoiding inference regarding recent demographic events (within the last hundred generations) using whole-genome data for fewer than 10 individuals or reduced-representation data for fewer than 100 individuals. The greater difficulty involved in inferring contemporary population size was reflected in generally much higher error rates for recent population sizes compared to historical sizes for most methods. In line with Beichman et al.’s recommendations, we observed the lowest error rates for the whole-genome data linkage method GONE, particularly when sample sizes were >25, and for momi2 and NeEstimator when using sample sizes >100. The Stairway Plot generally performed worse than momi2 for inferring recent size except when sample sizes were large (100). However, the Stairway Plot did outperform a number of other whole genome methods and accurately reconstructed an ∼100-fold decline over the past 100 years in Tasmanian devils (Patton et al. 2019).

### The utility of temporal versus contemporary data

Temporal sampling schemes contain specific information that contemporary-only schemes lack, in the form of both direct information on the genetic composition of past populations (which is leveraged by methods that provide point estimates of population size, such as the LD method implemented in NeEstimator) as well as information on the magnitude of genetic drift over time (used by the Jorde–Ryman method, Jorde and Ryman 2007). Our results; however, demonstrate that contemporary-only samples contain a substantial amount of information on changes in size over time as well, and it may not be necessary or sufficient to incorporate temporal data in order to accurately infer population sizes. This may be somewhat counterintuitive, as temporal data have been used extensively in the past for inferring population size changes (e.g., Ramakrishnan et al. 2005, Skoglund et al. 2014, Nunziata et al. 2017). Critically, however, when testing a method that can incorporate either contemporary or temporal schemes (momi2), we found that temporal data did not perform noticeably better compared to contemporary-only data when keeping the total number of samples constant and assuming the model was appropriately specified. A possible explanation of this pattern is that the additional information on rare alleles gained from sequencing twice the number of individuals in a contemporary-only sample. These alleles can be particularly informative for inferring recent changes in population size; rare alleles will be lost quickly in a bottleneck (Allendorf 1986).

NeEstimator using temporal data performed noticeably worse than other methods, and performance did not seem to be increased by increasing sample size. In contrast to our results, Nunziata and Weisrock (2018)found NeEstimator to be more accurate for detecting recent size changes, although they only assessed scenarios where the starting population size was on the smaller end of the range used here. The performance of this method is dependent on population size, and as most of our scenarios involved population declines the higher historic population sizes may have affected both. NeEstimator in particular requires a small but substantial proportion of the population to be sampled (∼1%; Marandel et al. 2019), and in cases where the population size is on the order of 10,000, our simulated datasets would not have had sufficient numbers of individuals. Sufficient historic sample sizes may be possible to obtain sometimes, but in many cases would be difficult to obtain for many species. It may, thus, be difficult to use NeEstimator to accurately infer historic population sizes for most populations, unless they were historically very small and isolated.

### Confounding factors

Both MAF filtering and the presence of singletons associated with sequencing error can cause extreme biases in estimates of contemporary population size for SFS-based methods. The loss of rare alleles is characteristic of a bottleneck (Allendorf 1986, Garza and Williamson 2001), and the application of a minor allele filter can create the illusion of a severe, recent bottleneck. An excess of rare alleles, on the other hand, is characteristic of a recent expansion (Keinan and Clark 2012), and the introduction of singleton errors could therefore lead to erroneous inference of an expansion. Interestingly, temporal sampling did seem to reduce the bias associated with minor allele filtering in our analyses, possibly because temporal data contain more explicit information on drift. Another explanation for this observation may be that, with smaller sample sizes per time point, applying a dataset-wide MAF cutoff will be less likely to remove truly rare alleles as the observed population allele frequencies are more affected by sampling variation and smaller sample sizes at each time point. We note that although we did not consider other sequencing artifacts that can have a substantial effect on the SFS (i.e. allelic dropout for RAD data; Heller et al. 2021), researchers should be aware of these as additional confounding factors in demographic inference. We also did not examine the effects of singletons on WGS methods. Singletons can be masked in Stairway Plot 2 (Liu and Fu 2020) and for GONE MAF has little effect on estimated population size (Novo et al. 2022), and as such these methods should be less sensitive to singletons and minor allele filtering, respectively. However, future work examining the effect of genotyping error could be worthwhile, especially since WGS studies in non-model species often use low-coverage WGS for which accurate genotype calling is difficult (Lou et al. 2021).

Historic population sizes are rarely stable over deeper time scales, and as such it is important for demographic inference methods to be robust to these more ancient changes. We simulated a 10-fold expansion or decline similar to a demographic change experienced by many organisms at the time of the last glacial maximum (Hewitt 2004). Encouragingly, the methods we examined seemed to be fairly robust to more ancient changes when inferring recent or historic sizes. Demographic reconstruction methods such as the Stairway Plot or GONE possess a built-in ability to infer these changes as they attempt to infer the entire demographic history of the population. Care must be taken with methods in which the user specifies the demographic model to fit, such as momi2, since these methods will only include ancient declines if the user includes them in the set of models to assess. If they are not included, then inferences of historic and recent size may be severely biased, as seen in our results. We also note that while we did not include recent population expansion in our set simulated scenarios, the signatures of expansion and decline are opposite (Beichman et al. 2018), and power to distinguish expanding populations from declining populations should be at least as high as power to distinguish expanding populations from stable populations. Accurately detecting recent expansions (in, e.g., invasive species) is also highly relevant to conservation and would be a worthwhile avenue for future research.

The genetic signal of demographic change accumulates on the scale of generations, and as such longer generation times (and therefore fewer generations elapsed) could severely reduce accuracy for inferring recent change. We did find lower accuracy for SFS-based methods when we increased generation time to 3 years. Organisms of conservation concern may have much longer generation times, and care should be taken to consider the number of generations since suspected declines. Nunziata and Weisrock (2018) found that declines were detectable when 10–20 generations had elapsed since the start of a decline. GONE seemed to perform fairly well even when only 10 generations had elapsed since the start of a decline, suggesting that using this method with whole genome data may be the best for inferring recent declines when generation times are longer. Before choosing a data type and method, researchers should consider the generation time of their study organisms and the number of generations that have elapsed since suspected changes in population size. If generation time is unknown (as it may be for some non-model species), researchers can attempt to estimate generation time for closely related species. Since the timing of estimated declines is scaled to generation time, uncertainty in generation time will mainly result in uncertainty in the timing of the change rather than the magnitude of change.

A number of factors that we did not examine here could potentially confound the inference of population size. We considered only a single panmictic population for simplicity. Inferring population size is less straightforward in structured populations and populations receiving migration (Neel et al. 2013, Orozco-terWengel 2016, Mazet et al. 2016). Model-based analyses can potentially include migration and population structure in their framework, although it would be important to sample all populations in that case. GONE seems to be robust to high levels of gene flow (in which case it infers a metapopulation-level estimate of size), but low levels of migration can distort estimates (Santiago et al. 2020). Researchers should be aware of any potential population structure when applying these methods.

The time scale on which whole genome data are informative for inferring recent change will depend on the frequency of recombination—specifically, recent recombination events are more likely to occur between alleles that are less tightly linked (McVean 2002). As such, long-range linkage data are necessary for inferring recent demography. While a reference genome is needed for providing the linkage information, our investigations suggest that the reference genome used does not need to be chromosome-scale. Specifically, reducing the size of known linkage groups to 5 cMs did not seem to meaningly affect inference with GONE, suggesting that even when using an incomplete draft genome, this method can provide reliable inference (Supplementary Fig. 12). Recombination rate variation could also potentially influence inferences of population size made using GONE. While recombination rate was fully determined by physical distance for our simulated datasets, recombination rate can vary substantially across the genome in real populations (Peñalba and Wolf 2020). When it is possible to construct accurate linkage maps, incorporating these maps in GONE and similar analyses would improve inference of recent population size.

In a recent review, Marchi et al. (2021) noted that whole genome data may not always be ideal for demographic inferences compared to reduced-representation data, since patterns of variation in whole genome data will be more influenced by non-stationary processes such as variation in recombination rates and selection across the genome that are difficult to model. It will be important to detect and account for these processes whenever possible while using whole genome data. GONE seems to be robust to selection (Novo et al. 2022) and can incorporate observed recombination rates across the genome rather than use a uniform rate (Santiago et al. 2020), meaning that this method could potentially surmount these obstacles presented by genome-scale data.

### Recommendations and future directions

Based on our results, we recommend different methods for inferring recent changes in population size depending on the samples and resources available. When contemporary whole-genome sequencing data can be collected from at least 50 samples and a reasonably complete draft genome is available, we recommend the linkage-based methods implemented in GONE. These methods appear powerful and accurate across a wide-range of demographic scenarios. In contrast, we recommend the SFS-based momi2 when linkage information and whole-genome data are not available. In particular, we recommend serial sampling with momi2 to help reduce the impacts of model misspecification or genotyping error, both of which are difficult to fully avoid. Care must be taken; however, to ensure that the SFS used for inference with momi2 accurately represents the full SFS (including rare alleles) in the population of interest. While NeEstimator performed relatively poorly in our tests, it could be useful when historical and contemporary samples are available and when an appreciable fraction of the population (1%) has been sampled at each time point.

There are currently gaps in methods that can incorporate whole-genome data with historical samples and in methods that can combine SFS and linkage information. ABC and machine learning methods could bridge this gap (Beichman et al. 2018, Schrider and Kern 2018, Sanchez et al. 2021), and they represent promising approaches for integrating multiple data types. Moving forward, it will be important to evaluate these new methods under a wide-range of scenarios and data types to determine how useful they are for inferring recent size changes.

## Supplementary Material

icac144_Supplemental_Files

## Data accessibility

Scripts used to conduct simulations and plot results can be found at https://github.com/philippinespire/PIRE_TemporalSims_HPC.
