# Supplementary material for: Simulation-Based Evaluation of Methods, Data Types, and Temporal Sampling Schemes for Detecting Recent Population Declines
Source: Integr Comp Biol. 2022 Sep 14;62(6):1849–63. doi: 10.1093/icb/icac144 (PMC9801984; doi:10.1093/icb/icac144)
Supplement: icac144_Supplemental_Files [file icac144_supplemental_files.zip › Revised_Supplemental_Figures_Tables.pdf]

Supplemental Figure 1. Distribution of infinite estimates for (a) $N_{e,H}$ and (b) $N_{e,C}$ from NeEstimator. X-axis gives sample sizes used and y-axis gives simulated $N_{E,H}$ value.

(a)

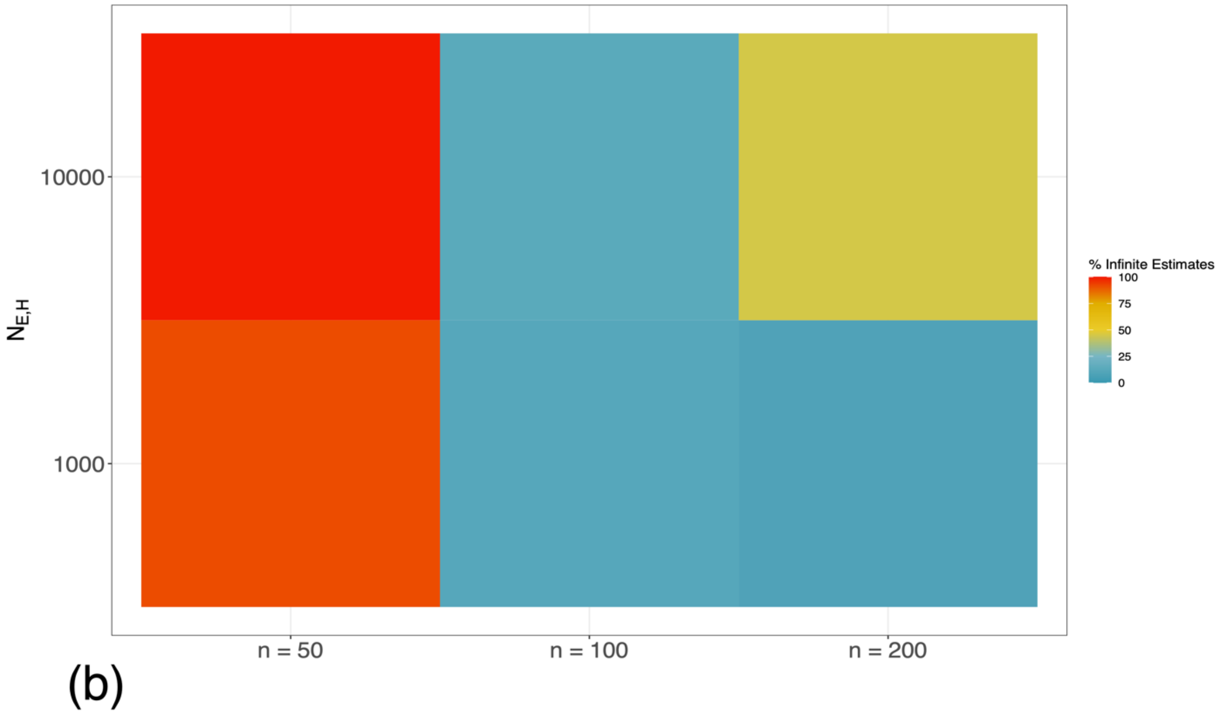

(b)

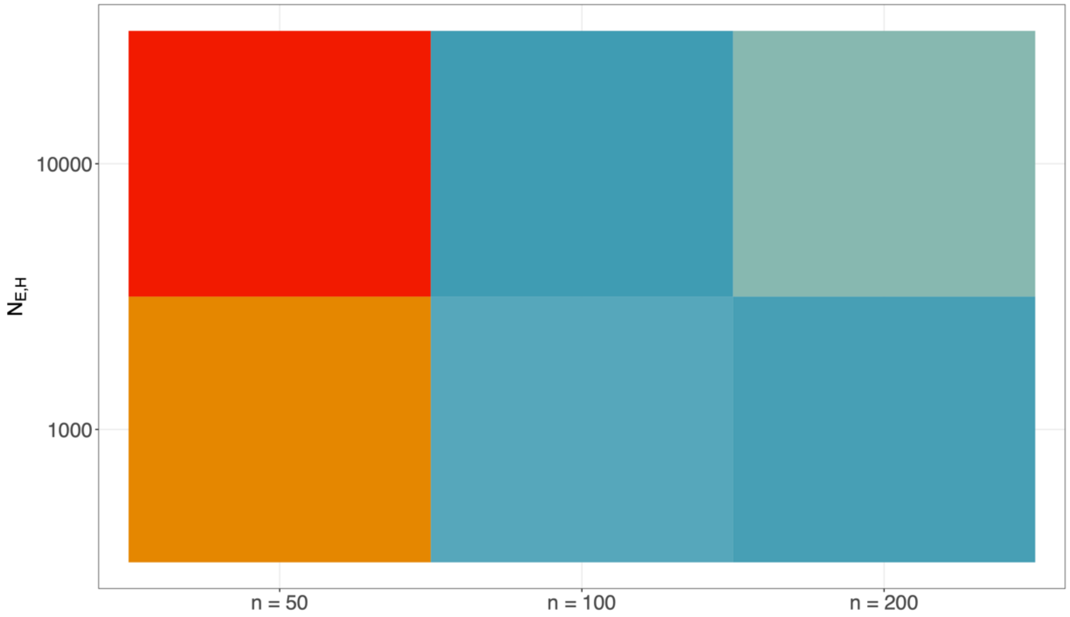

Supplementary Figure 2. Violin plots showing accuracy for estimating (a) $N_{e,H}$ and (b) $N_{e,C}$ combined over all demographic scenarios and historic population sizes. Ratio of estimated to true N_E is plotted on a $\log_{10}$ scale. Perfect agreement between simulated and estimated values is shown as a 1:1 dotted line.

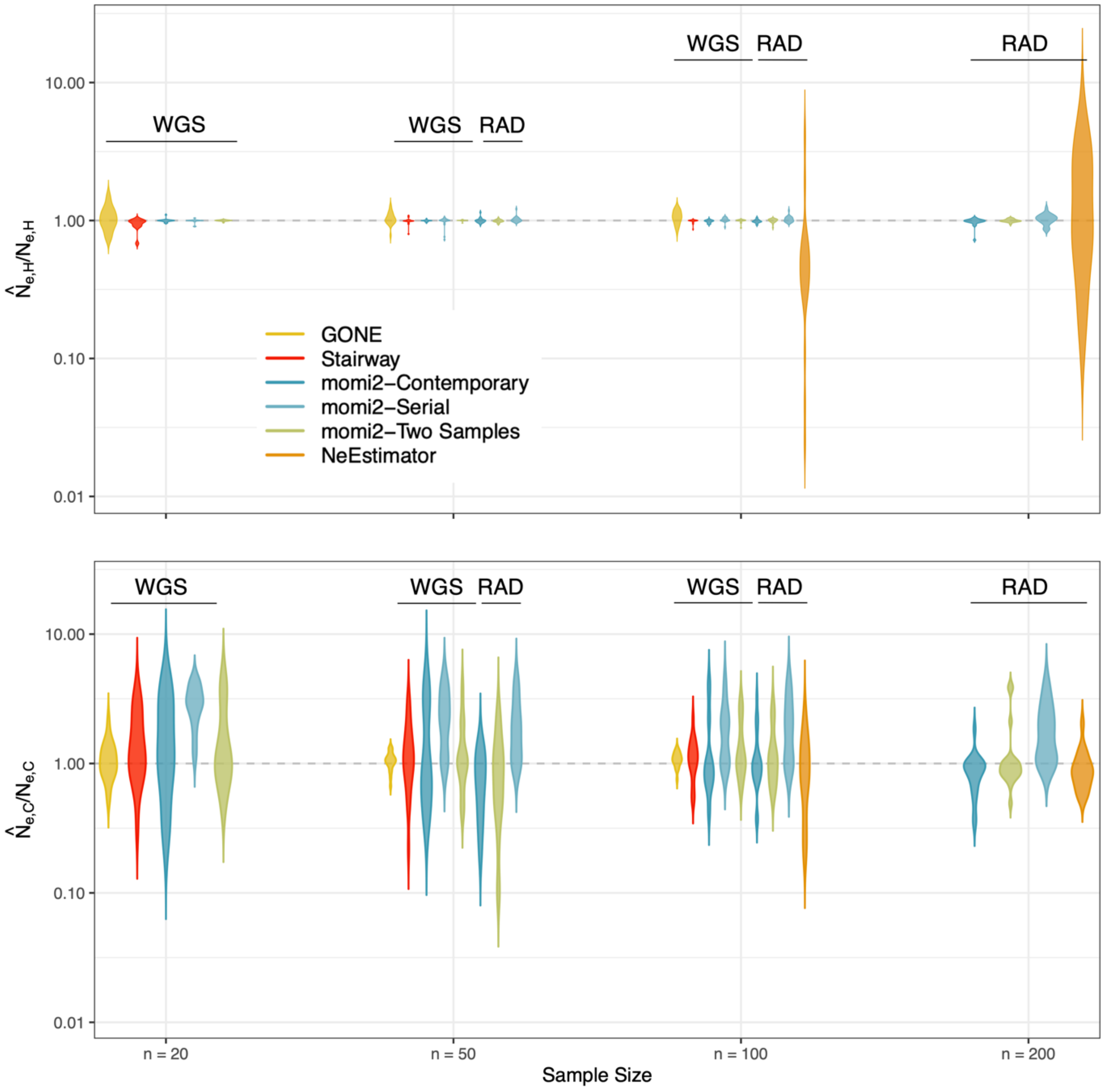

Supplemental Figure 3. Distribution of estimates for historic population size ($N_{e,H}$) broken down by each method, data type, decline scenario, and historic population size. Ratio of estimated to true N_e is plotted on a $\log_{10}$ scale. Perfect agreement between simulated and estimated values is shown as a 1:1 dotted line.

(a)

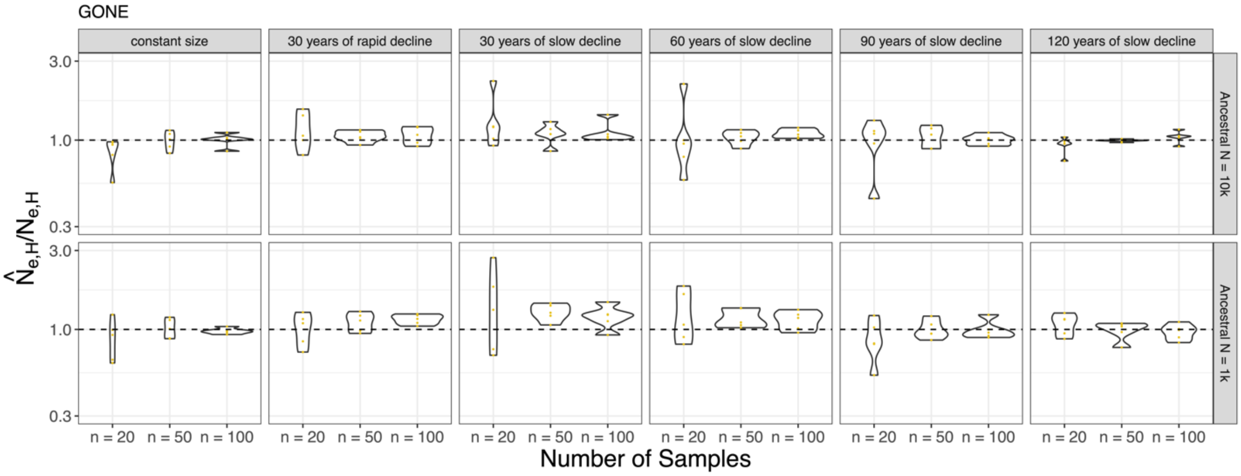

(b)

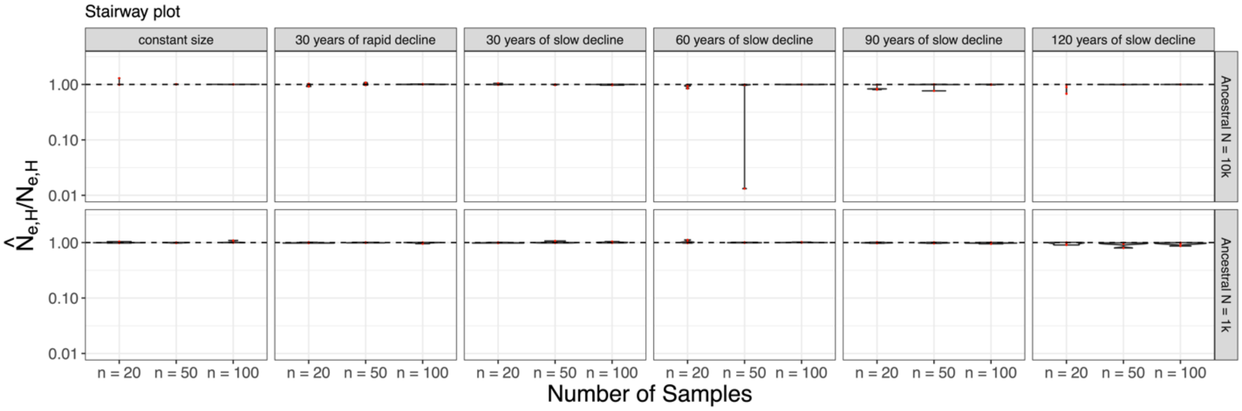

(c)

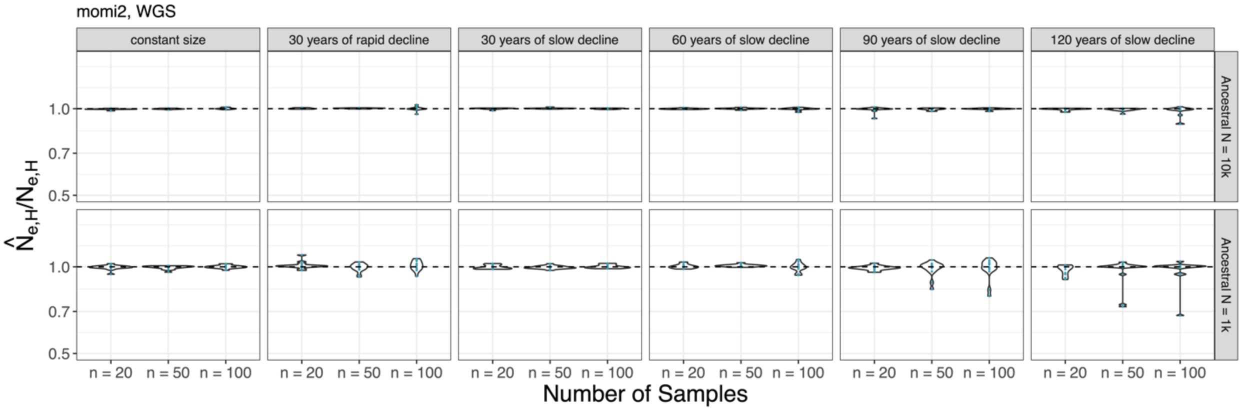

(d)

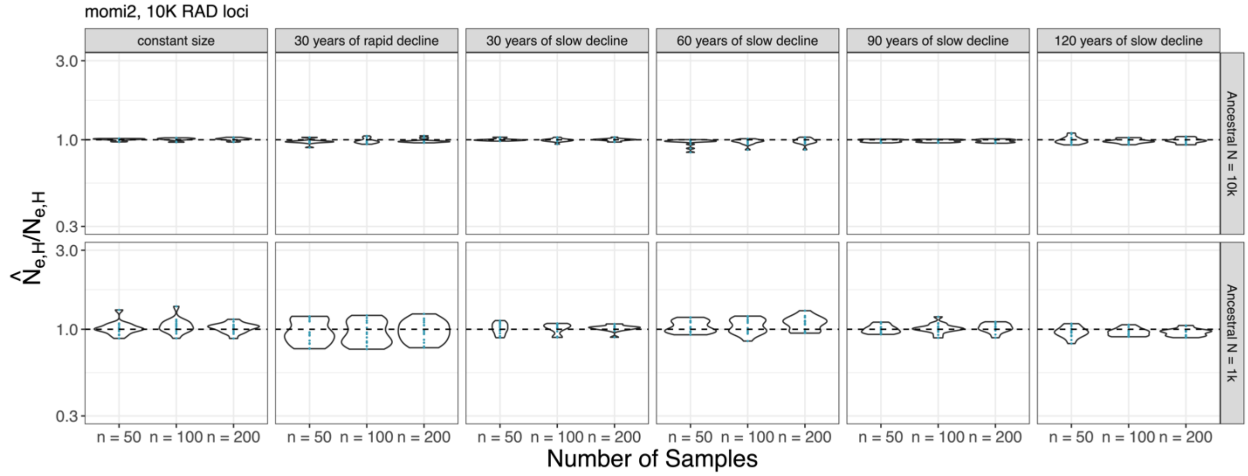

momi2, 10K RAD loci
constant size
30 years of rapid decline
30 years of slow decline
60 years of slow decline
90 years of slow decline
120 years of slow decline
Ancestral N = 10k
Ancestral N = 1k
3.0
1.0
0.3
$\hat{N}_{e,H}/N_{e,H}$
n = 50
n = 100
n = 200
Number of Samples

(e)

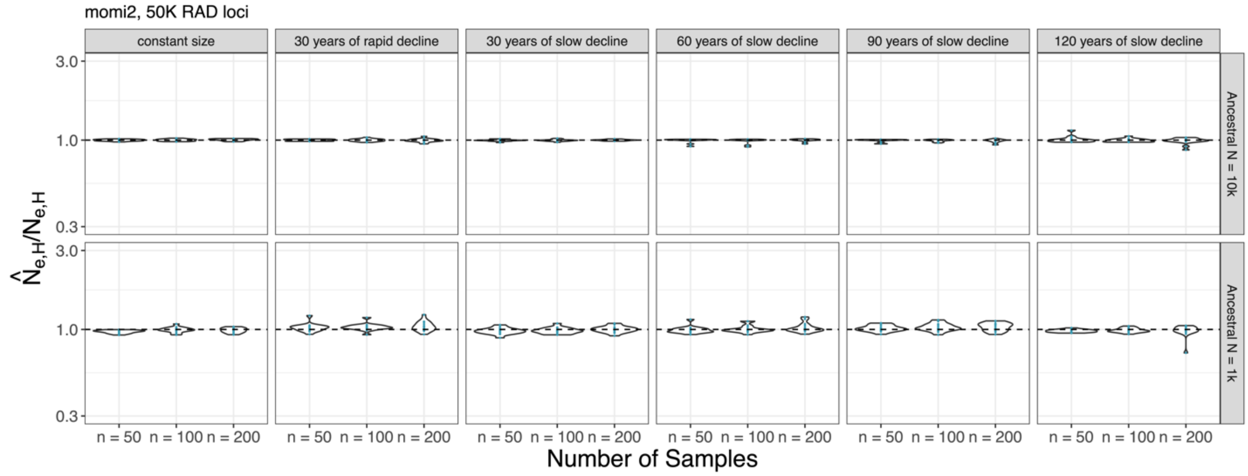

momi2, 50K RAD loci
constant size
30 years of rapid decline
30 years of slow decline
60 years of slow decline
90 years of slow decline
120 years of slow decline
Ancestral N = 10k
Ancestral N = 1k
3.0
1.0
0.3
$\hat{N}_{e,H}/N_{e,H}$
n = 50
n = 100
n = 200
Number of Samples

(f)

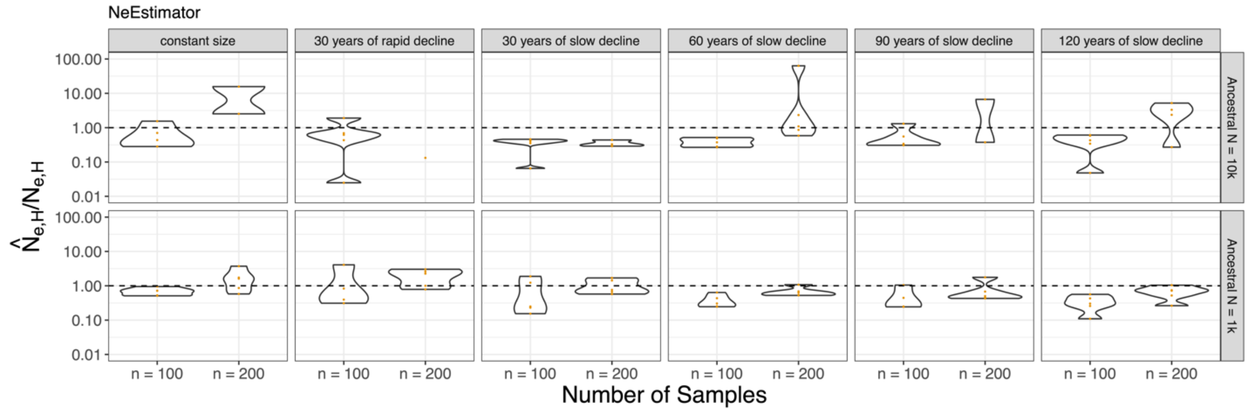

NeEstimator
constant size
30 years of rapid decline
30 years of slow decline
60 years of slow decline
90 years of slow decline
120 years of slow decline
Ancestral N = 10k
Ancestral N = 1k
100.00
10.00
1.00
0.10
0.01
$\hat{N}_{e,H}/N_{e,H}$
n = 100
n = 200
Number of Samples

Supplemental Figure 4. Distribution of estimates for contemporary population size ($N_{e,C}$) broken down by each method, data type, decline scenario, and historic population size. Ratio of estimated to true N_e is plotted on a $\log_{10}$ scale. Perfect agreement between simulated and estimated values is shown as a 1:1 dotted line.

(a)

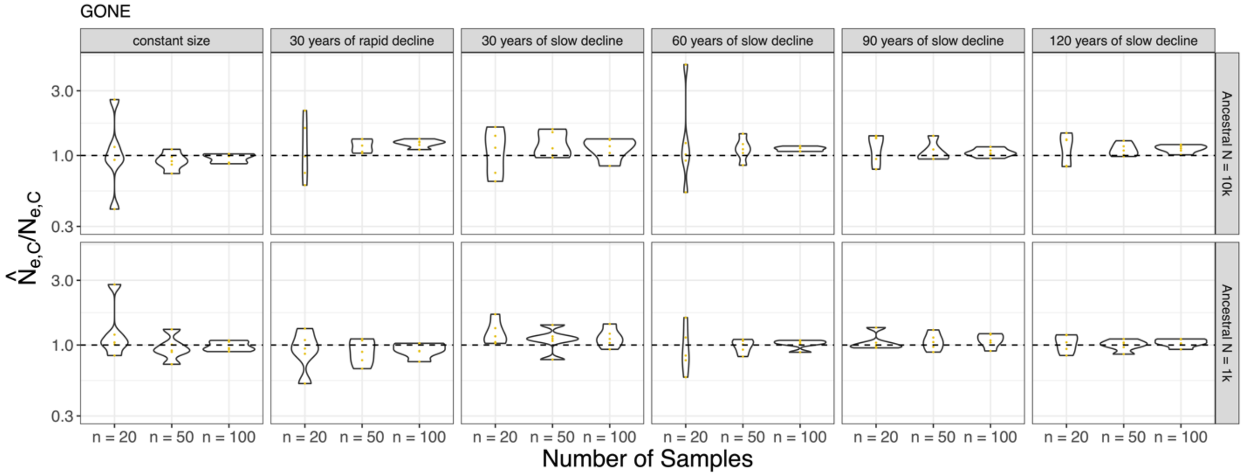

(b)

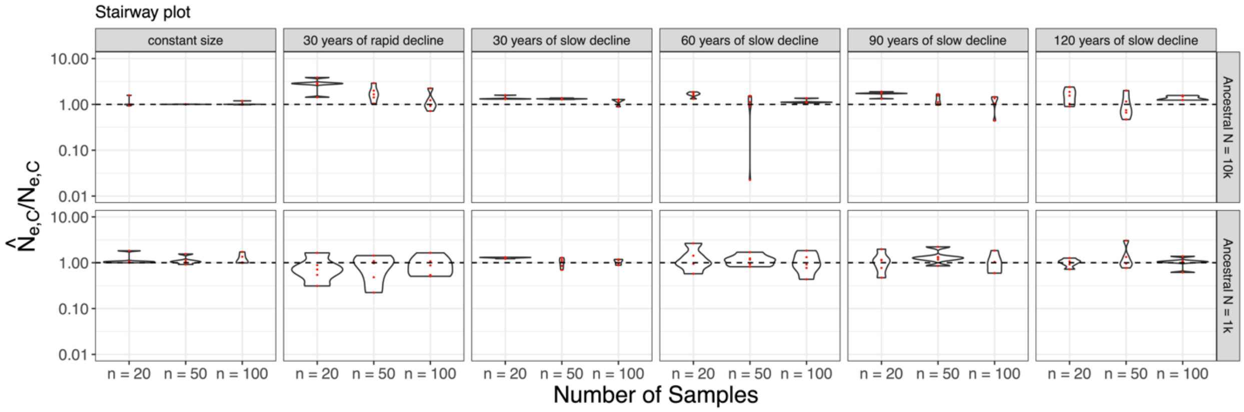

(c)

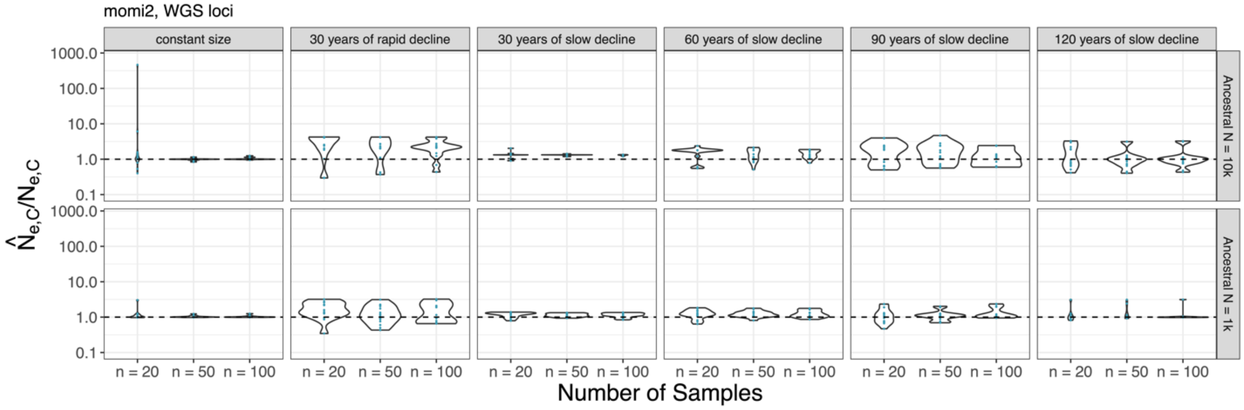

(d)

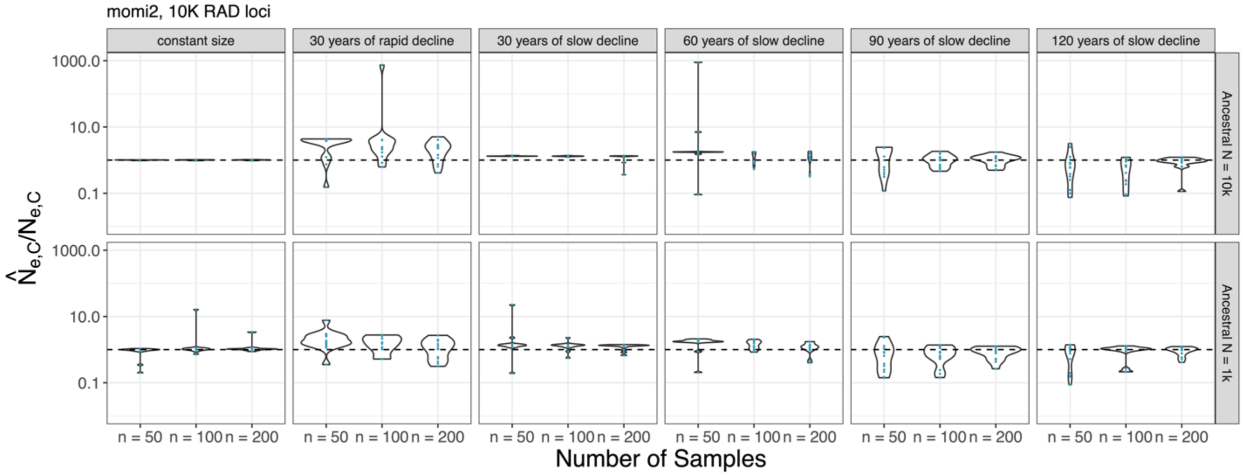
momi2, 10K RAD loci
constant size
30 years of rapid decline
30 years of slow decline
60 years of slow decline
90 years of slow decline
120 years of slow decline
Ancestral N = 10k
Ancestral N = 1k
1000.0
10.0
0.1
$\hat{N}_{e,C}/N_{e,C}$
n = 50
n = 100
n = 200
Number of Samples

(e)

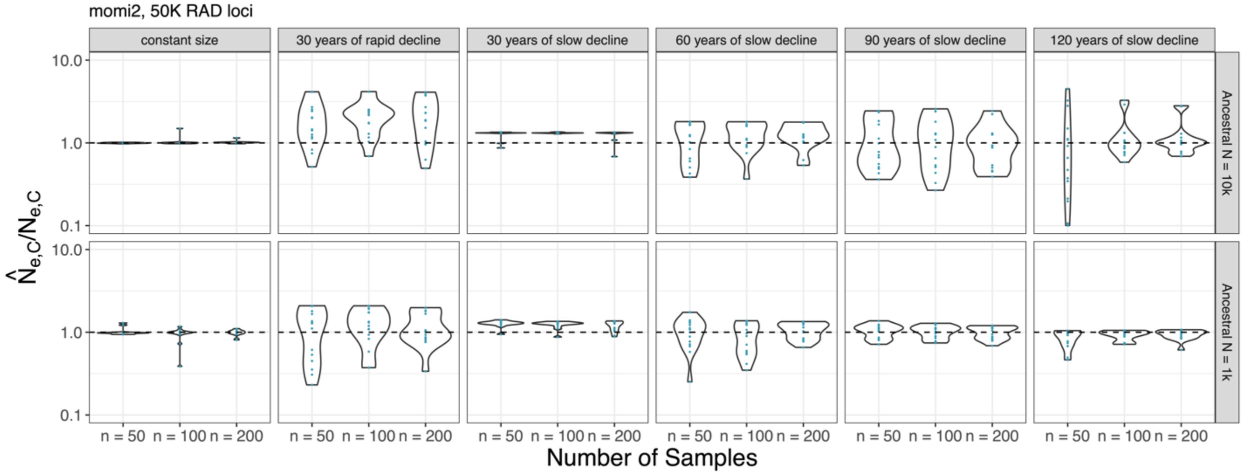
momi2, 50K RAD loci
constant size
30 years of rapid decline
30 years of slow decline
60 years of slow decline
90 years of slow decline
120 years of slow decline
Ancestral N = 10k
Ancestral N = 1k
10.0
1.0
0.1
$\hat{N}_{e,C}/N_{e,C}$
n = 50
n = 100
n = 200
Number of Samples

(f)

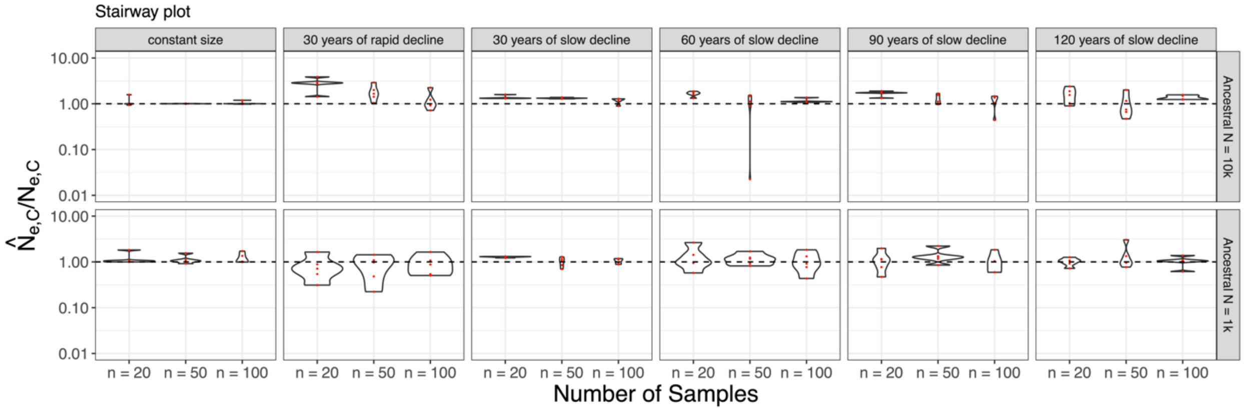
Stairway plot
constant size
30 years of rapid decline
30 years of slow decline
60 years of slow decline
90 years of slow decline
120 years of slow decline
Ancestral N = 10k
Ancestral N = 1k
10.00
1.00
0.10
0.01
$\hat{N}_{e,C}/N_{e,C}$
n = 20
n = 50
n = 100
Number of Samples

Supplemental Figure 5. Full model selection accuracy results for momi2. Demographic scenarios are defined by the length of the decline (x number of years, starting x years before the contemporary time point) and the speed of the decline (λ, equivalent to the ratio of number of offspring in a given year divided by the number of offspring in the following year). Percent of simulations in which the correct demographic model had the lowest AIC are shown for datasets with ten thousand RAD loci (10K RAD), fifty thousand RAD loci (50K RAD), or whole genome data (WGS).

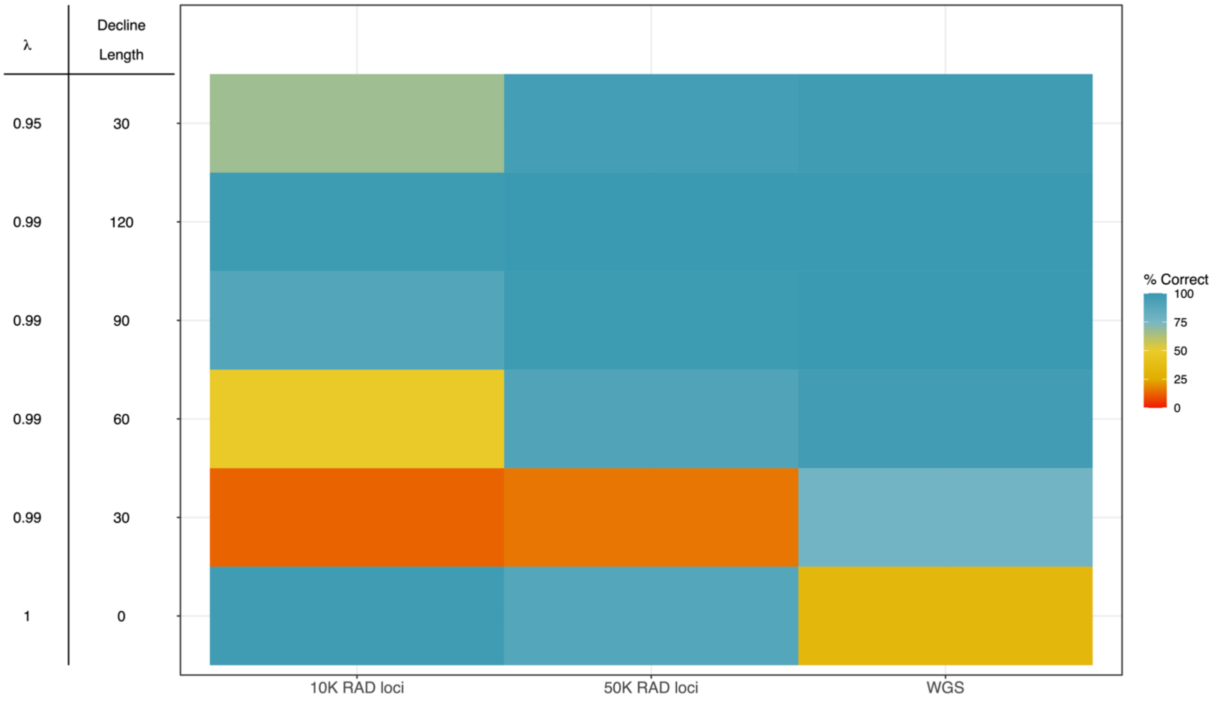

Supplemental Figure 6. Power to detect declines for the G3 life history scenario.

(a) WGS data, $n = 100$.

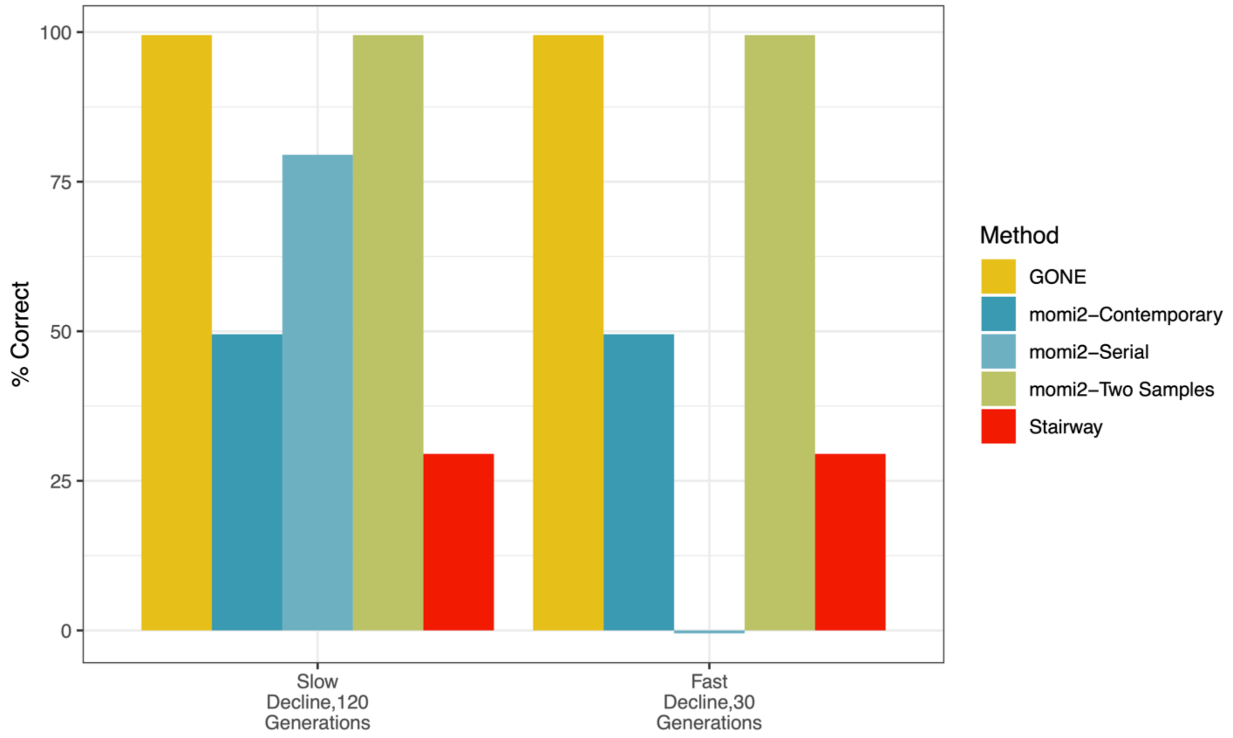

(b) RAD data, $n = 200$

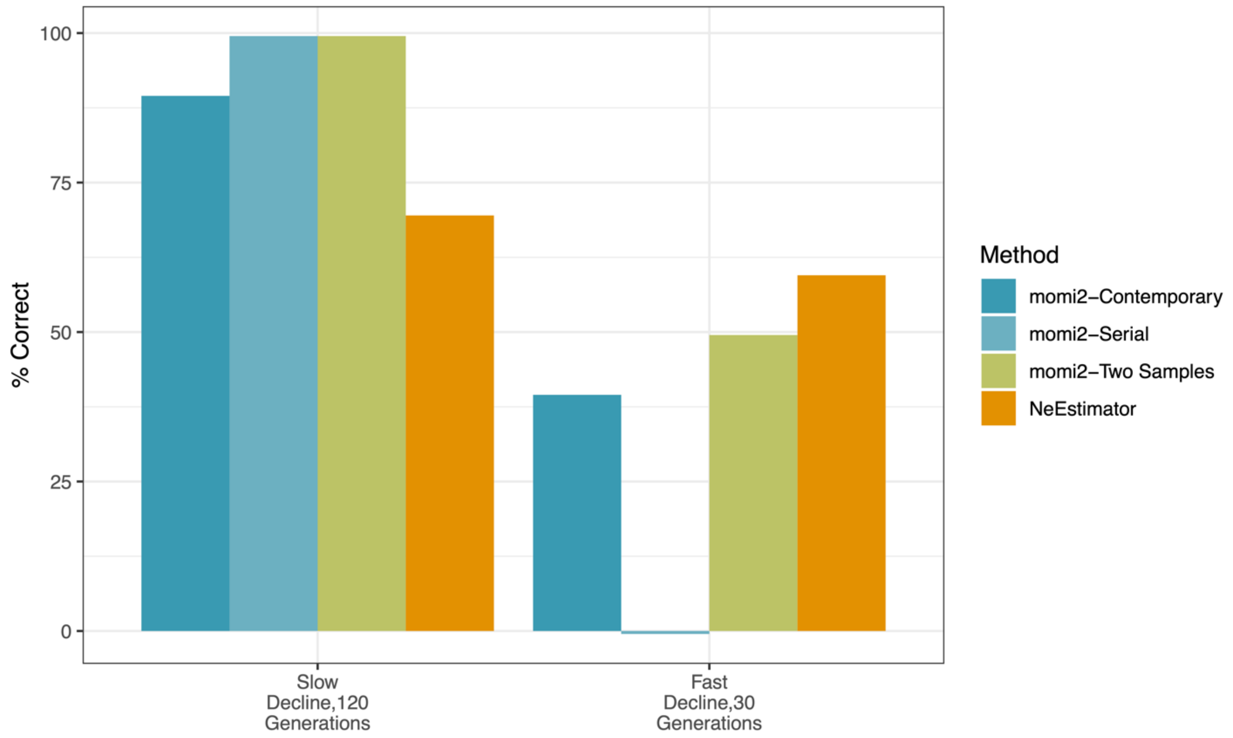

Supplemental Figure 7. Precision and accuracy for momi2 inferences across different sampling schemes and generation times. Black violins correspond to $N_{E,H}$, while white violins show $N_{E,C}$. Ratio of estimated to true N_E is plotted on a $\log_{10}$ scale. Perfect agreement between simulated and estimated values is shown as a 1:1 dotted line.

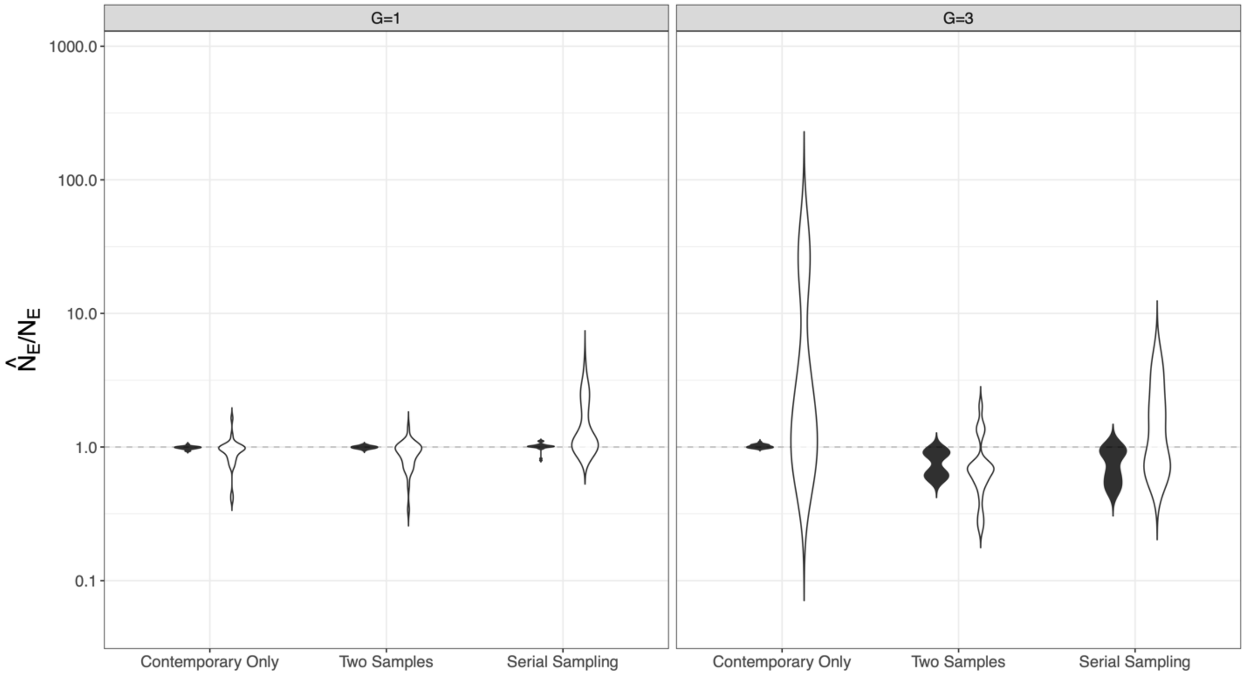

Supplemental Figure 8. Momi2 model selection under (a) ancestral bottleneck and (b) ancestral expansion scenarios. Demographic scenarios are defined by the length of the decline (x number of years, starting x years before the contemporary time point) and the speed of the decline (λ, equivalent to the ratio of number of offspring in a given year divided by the number of offspring in the following year). Percent of simulations in which the correct demographic model had the lowest AIC are shown for datasets with ten thousand RAD loci (10K RAD), fifty thousand RAD loci (50K RAD) or whole genome data (WGS).

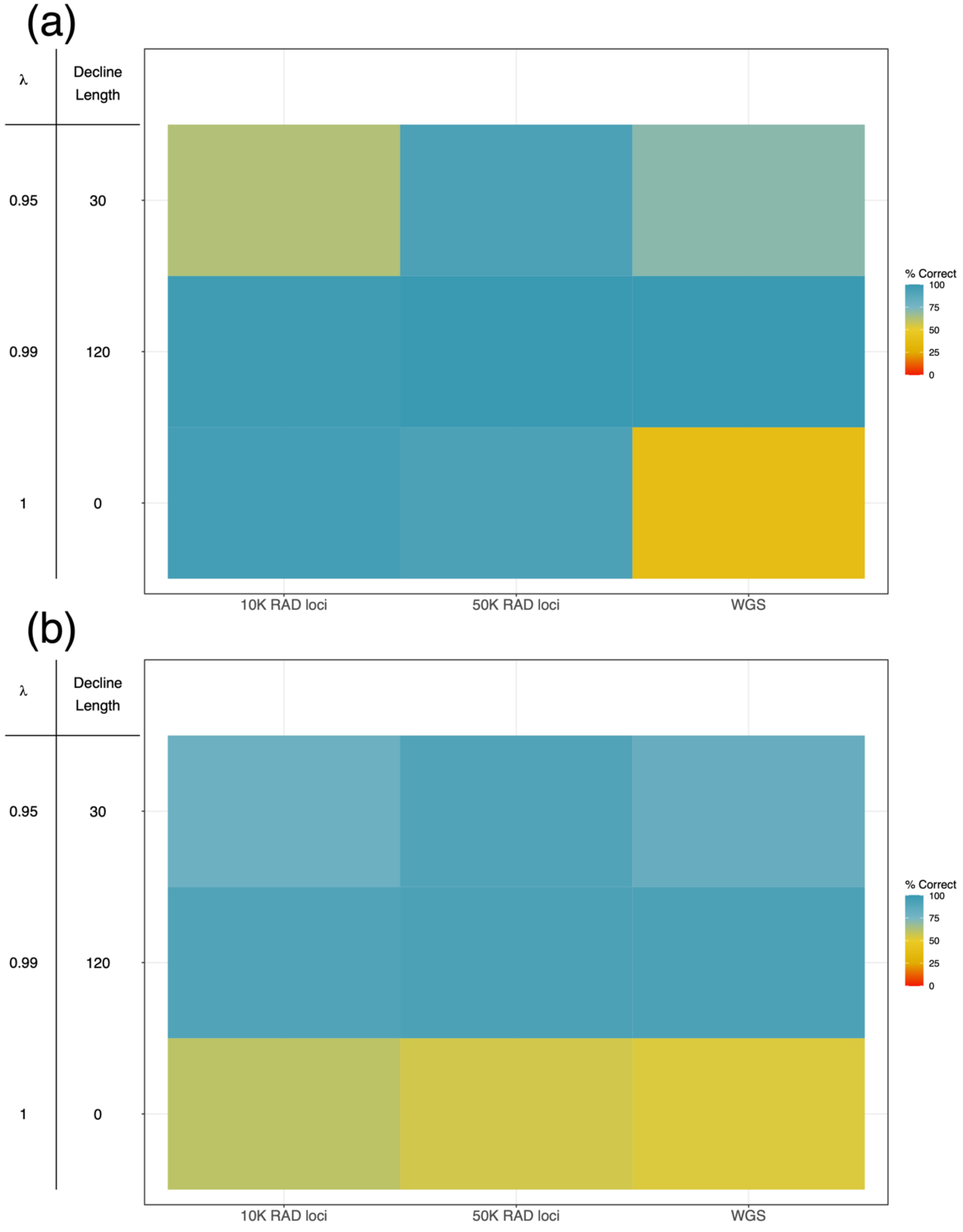

Supplementary Figure 9. Effect of model mis-specification on momi2 accuracy and precision for estimating (a) $N_{e,H}$ and (b) $N_{e,C}$ under ancestral bottleneck and expansion scenarios. Inferences made using the full set of potential models are shown in white, while inferences made while assuming there were not any ancestral size change (i.e., model misspecification) are shown in red. Ratio of estimated to true N_e is plotted on a log_{10} scale. Perfect agreement between simulated and estimated values is shown as a 1:1 dotted line.

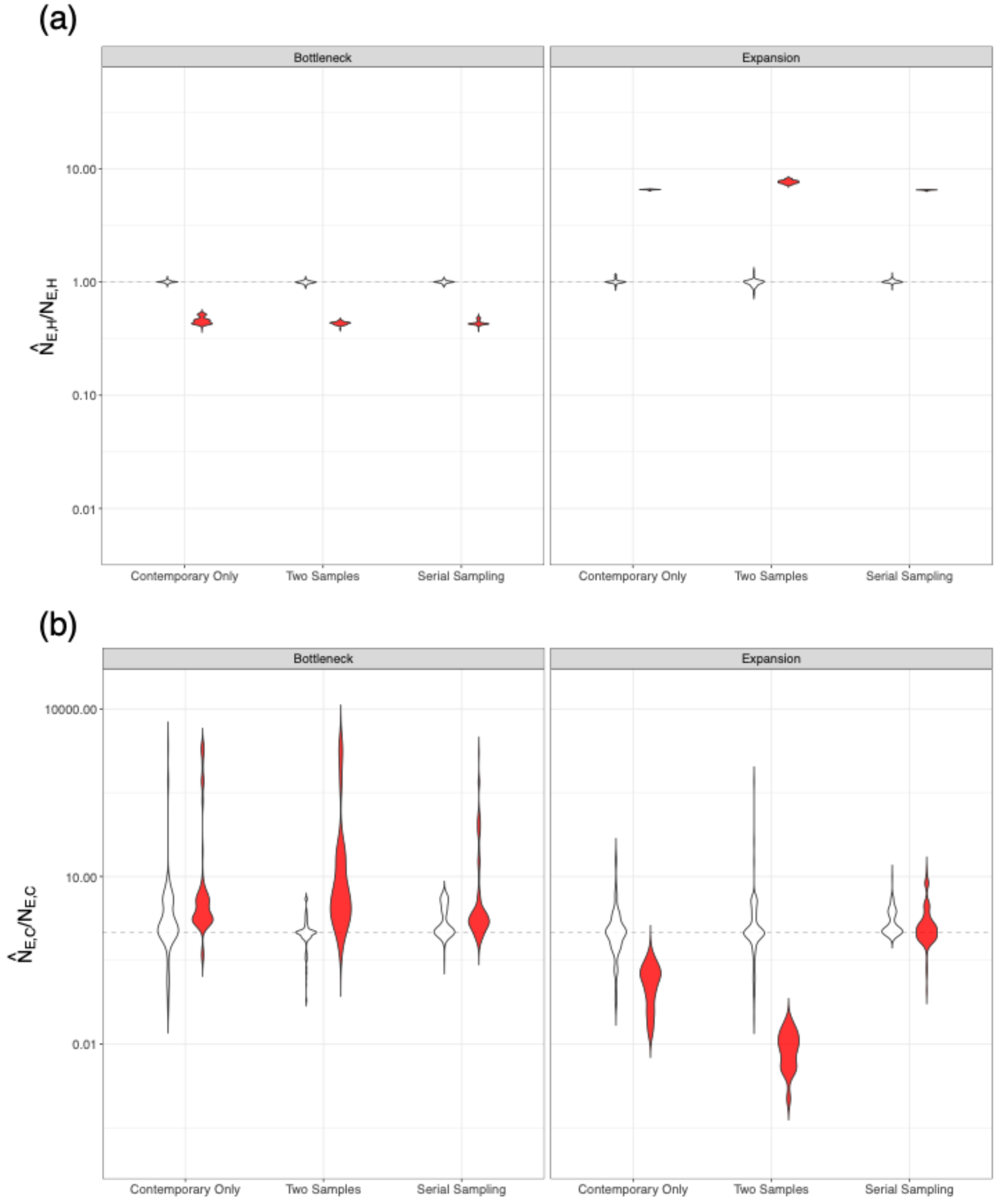

Supplemental Figure 10. Effects of adding singleton errors to the contemporary-only dataset on inferences with momi2. Black-and-white boxplots correspond to inferences for $N_{e,H}$, while gray-and-white boxplots correspond to inferences for $N_{e,C}$.

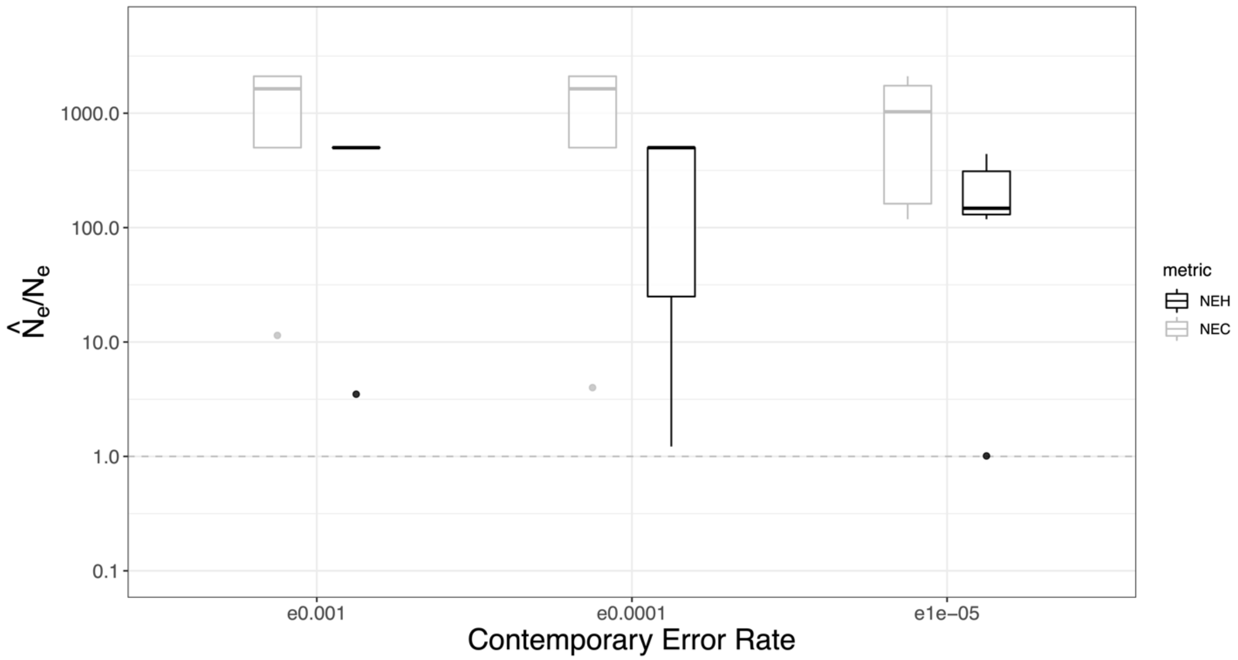

Supplemental Figure 11. Precision for momi2 inferences across different temporal sampling and minor allele filtering schemes. Black violins correspond to $N_{e,H}$, while white violins show $N_{e,C}$. Ratio of estimated to true N_E is plotted on a $\log_{10}$ scale. Perfect agreement between simulated and estimated values is shown as a 1:1 dotted line.

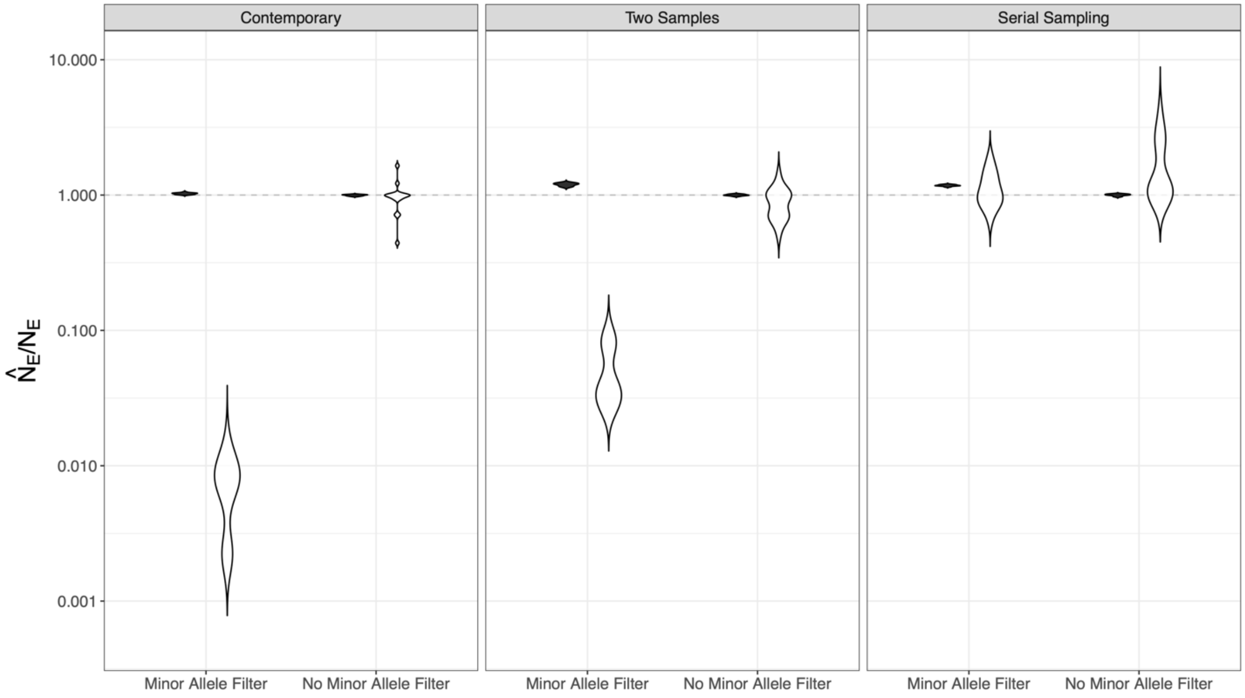

Supplemental Figure 12. While GONE relies on detailed linkage information for inferring changes in NE and has previously been evaluated assuming a chromosome-level reference genome for providing this information, draft-quality reference genomes for non-model organisms are often incomplete and fragmented (Brandies et al. 2019). To assess the potential effects of using a draft reference genome for inference using GONE, we altered the ped/map files to simulate mapping to a somewhat fragmented genome with 150 scaffolds 5Mb in length each (retaining all linkage information within these scaffolds but assuming no linkage across scaffolds) and re-ran GONE on this altered dataset. We also simulated an even more fragmented genome with 1Mb scaffolds using a similar method. Since GONE can accept a maximum of 199 linkage groups, in this scenario we also randomly retained 199 scaffolds (discarding the rest of the data) and re-ran GONE.

Estimation accuracy for $N_{e,H}$ and $N_{e,C}$ with GONE using data modified to reflect a moderate-quality reference genome assembly (150x5Mb scaffolds) was similar overall to estimation using a full chromosome-level reference genome assembly (25x30 Mb chromosomes; Figure 7). In this case, the convergence between these two estimates is likely due to the fact that GONE will not use all pairs of loci, with the cutoff for our analyses set to 0.05 centiMorgans; as such, the 25x30Mb and 150x5 Mb schemes may be providing nearly the same input information. However, accuracy for both parameters decreased substantially when data from a lower-quality draft genome assembly (199x1Mb scaffolds) were used (Figure 8).

Figure 12. Effects of genome assembly scheme on inference using GONE. Results for $N_{E,H}$ are shown in black and results for $N_{e,C}$ are shown in white. Ratio of estimated to true N_e is plotted on a log_{10} scale. Perfect agreement between simulated and estimated values is shown as a 1:1 dotted line.

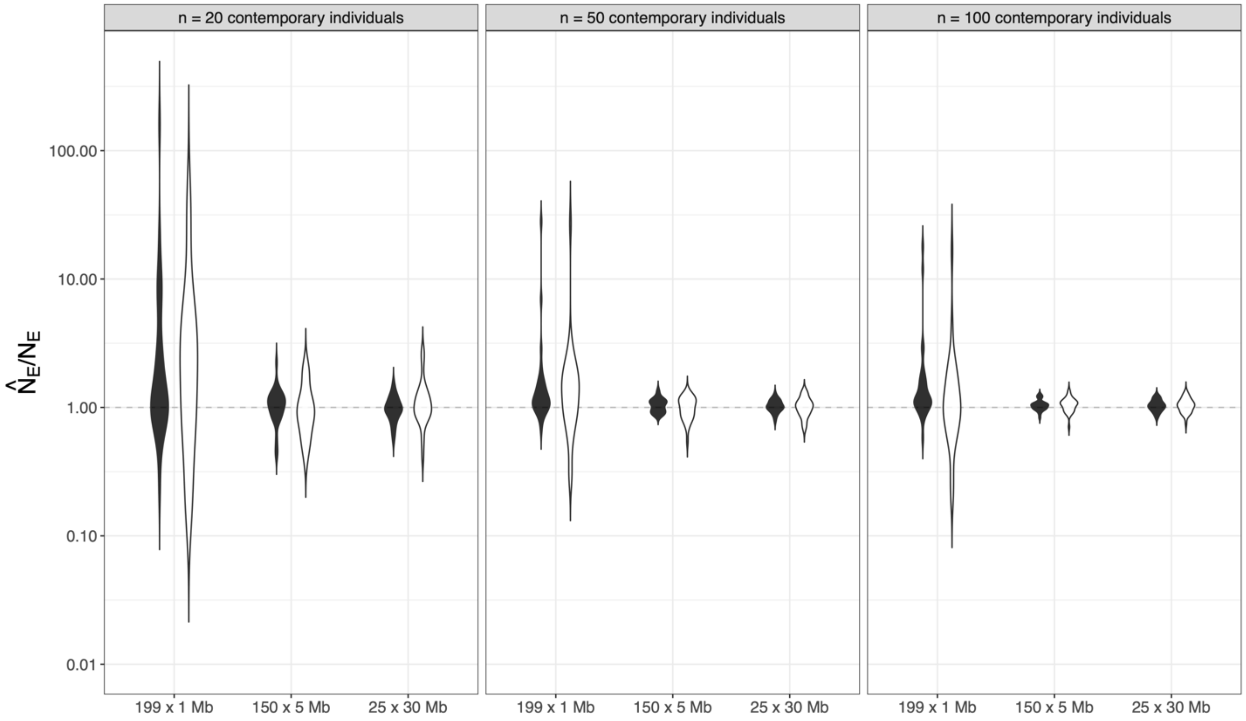

Supplementary Table 1. Age-mortality relationship for simulations with generation time = 3.

**Age**	**Mortality rate**
1	0.733
2	0.2
3	0.2
4	0.2
5	0.2
6	0.36
7	0.6
8	0.8
9	1
